# Vitamin D-Related Signaling and Epigenetic Regulation: Evidence from Experimental, Observational, and Interventional Studies

**DOI:** 10.3390/ph19060906

**Published:** 2026-06-08

**Authors:** Hanna Kozłowska, Edyta Cichocka, Sylwia Barbara Górczyńska-Kosiorz, Janusz Gumprecht

**Affiliations:** Department of Internal Medicine, Diabetology and Nephrology, Faculty of Medical Sciences in Zabrze, Medical University of Silesia, 40-055 Katowice, Poland

**Keywords:** vitamin D, cholecalciferol, 1,25(OH)_2_D, calcitriol, epigenomics, DNA methylation, histone modifications, non-coding RNA

## Abstract

The active vitamin D metabolite, 1,25-dihydroxycholecalciferol [1,25(OH)_2_D], exerts its biological effects through binding to the vitamin D receptor (VDR), a ligand-activated transcription factor regulating the expression of genes involved in calcium and phosphate homeostasis, immune modulation, and cell proliferation and differentiation. In addition to direct transcriptional regulation, 1,25(OH)_2_D signaling also involves epigenetic mechanisms. A total of 90 studies were included in this narrative review, comprising experimental studies (*n* = 45), observational studies (*n* = 17), population-based studies (*n* = 8), interventional studies (*n* = 15), and mixed-design studies (*n* = 5). Experimental studies in cell cultures and animal models demonstrate that 1,25(OH)_2_D may affect several major epigenetic regulatory pathways, including chromatin remodeling, DNA methylation, histone modifications, and the expression of non-coding RNAs, particularly microRNAs. Preclinical evidence suggests that the epigenetic actions of 1,25(OH)_2_D are involved in metabolic regulation, immune responses, bone development, fibrotic processes, carcinogenesis, ageing, and fetal programming. However, evidence from observational studies and randomized controlled trials remains limited and inconclusive. Some studies have reported alterations in miRNA expression, methylation of selected loci, and epigenetic age markers. The clinical relevance of 1,25(OH)_2_D–mediated epigenetic regulation has not yet been fully established. The interpretation of available findings is limited by substantial heterogeneity in study populations, exposure and intervention protocols, environmental factors, interindividual variability in response to vitamin D supplementation associated with genetic polymorphisms and methylation status, and the restricted range of analyzed cell types. This subject requires randomized controlled trials integrating molecular endpoints with clinically relevant outcomes.

## 1. Introduction

Vitamin D has been referred to as a vitamin, a nutrient, and a hormone. However, the term vitamin D is often used inaccurately to describe a broad group of secosteroids, despite the fact that these compounds differ substantially in their structure, biological activity, and physiological roles. This oversimplification can lead to ambiguity in both scientific communication and the interpretation of experimental and clinical data. The challenge in establishing a consistent nomenclature arises primarily from the complexity of vitamin D synthesis, metabolism, and the wide range of its metabolites and derivatives [1].

Cholecalciferol, through its active metabolite, 1,25-dihydroxycholecalciferol [1,25(OH)_2_D], is classically involved in the regulation of calcium and phosphate homeostasis and bone metabolism, and its deficiency is a well-established cause of rickets and osteomalacia [2]. Over time, the role of 1,25(OH)_2_D has been expanded beyond skeletal health, with accumulating evidence demonstrating its involvement in a wide range of physiological processes, including the regulation of cell proliferation, differentiation, and adhesion, angiogenesis, metabolism, and innate and adaptive immune responses [2,3].

Alongside the discovery of extra-skeletal molecular mechanisms of 1,25(OH)_2_D action in experimental studies, an increasing body of evidence from population-based studies has suggested associations between low serum concentrations of 25-hydroxyvitamin D [25(OH)D] and an elevated risk of numerous conditions. These include infectious diseases (such as sepsis, pulmonary infections, tuberculosis, and COVID-19), autoimmune disorders (including psoriasis, type 1 diabetes, multiple sclerosis, and rheumatoid arthritis), cardiovascular diseases, metabolic syndrome, and cancer [2,4]. However, randomized controlled trials have thus far yielded inconclusive results regarding a causal relationship between vitamin D supplementation and clinical outcomes, such as reduced disease incidence or mortality [2,5,6,7]. It has been suggested that the beneficial effects of supplementation may be largely confined to individuals with vitamin D deficiency [2].

At the molecular level, the physiological actions of cholecalciferol are primarily mediated through the regulation of target gene expression by the vitamin D receptor (VDR) upon activation by 1,25(OH)_2_D [8]. The broad spectrum of 1,25(OH)_2_D effects is thought to result from the widespread expression of VDR across diverse human cell and tissue types, the presence of numerous cell-specific VDR binding sites throughout the genome [9], and the receptor’s ability to interact and cooperate with other nuclear proteins and transcription factors [10].

In recent years, increasing attention has been directed toward the epigenetic aspects of vitamin D signaling, which may represent a critical determinant of its biological effects. Vitamin D not only influences cellular function through epigenetic mechanisms but is itself subject to epigenetic regulation [11]. The aim of this narrative review is to summarize current evidence on the epigenetic mechanisms through which 1,25(OH)_2_D contributes to the regulation of physiological and pathological processes, based on findings from experimental studies, including cell line and animal models, as well as clinical and population-based studies on vitamin D deficiency and supplementation. Additionally, the review highlights the importance of interindividual variability in vitamin D responsiveness, as reflected by the vitamin D response index.

## 2. Methodology

A literature search covering studies published between 2010 and 2026 was conducted in the PubMed and Google Scholar databases in January, February, and May 2026 using the following keywords: “vitamin D supplementation”, “25(OH)D”, “1,25(OH)_2_D”, “calcitriol”, “epigenetics”, “DNA methylation”, “histone modification”, “non-coding RNA”, “VDR”, and “vitamin D response”. Studies addressing epigenetic aspects of vitamin D were selected, with particular focus on DNA methylation, histone modifications, and non-coding RNAs (miRNA and lncRNA). The review encompassed both experimental research examining the direct impact of 1,25(OH)_2_D on chromatin remodeling and population-based studies exploring associations between 25(OH)D serum levels and epigenomic patterns, as well as clinical trials assessing epigenetic outcomes of supplementation. Studies examining potential epigenetic mechanisms associated with the effects of vitamin D supplementation, particularly in the context of metabolic disorders, cancer, bone health, and aging, were included. In addition, genes and signaling pathways involved in immunomodulation, carcinogenesis, and metabolic regulation were analyzed to provide a broader perspective on the interactions between 1,25(OH)_2_D, the epigenome, and clinical phenotypes.

Special attention was given to the epigenetic regulation of the vitamin D axis, including genes essential for vitamin D metabolism and function, such as *VDR*, *CYP27B1*, *CYP24A1*, *CYP2R1*, and *GC*. The selection strategy further considered interindividual variability in response to supplementation by incorporating studies on genetic polymorphisms within vitamin D axis genes and their interaction with epigenetic mechanisms that may influence supplementation efficacy and therapeutic relevance.

Given the narrative nature of this review, it does not include all available studies and evidence.

## 3. Epigenomics

Epigenomics is the study of chromatin modifications that influence gene expression without altering the DNA sequence. These modifications can be transient or stable and, in some cases, heritable [12].

Chromatin is a dynamic complex composed of genomic DNA, histone proteins, DNA-binding factors, and RNA. Its fundamental structural unit is the nucleosome, consisting of DNA wrapped around an octamer of histone proteins [13]. Chromatin exists in two forms: tightly packed, transcriptionally inactive heterochromatin, and loosely organized, transcriptionally accessible euchromatin [14]. Key modifications influencing chromatin activity include DNA methylation, histone modifications [15], 3-dimensional chromatin organization [16] and the action of non-coding RNAs [17].

DNA methylation is primarily mediated by DNA methyltransferases (DNMTs) and ten-eleven translocation (TET) proteins [18]. DNA methylation can directly inhibit transcription factor binding or recruit repressive factors [19]. Histones undergo a variety of post-translational modifications regulated by enzymes such as histone acetyltransferases (HATs) and histone deacetylases (HDACs) [18,20]. Chromatin activity, and therefore gene expression, is also modulated by 3-dimensional organization into hierarchical loops dividing genome into topologically associating domains (TADs) [21,22]. Although the majority of the genome is transcribed, only 1–2% encodes proteins [19], while the remaining portion of the transcriptome consists of non-coding RNAs [23], including microRNAs (miRNAs), which exert their regulatory functions, among others, by inhibiting translation and promoting mRNA degradation [24], and long non-coding RNAs (lncRNAs), which modulate chromatin structure and regulate transcriptional and post-transcriptional processes [25,26,27].

In contrast to the genome, the epigenome is dynamic and responsive to environmental stimuli, including vitamin D via its active metabolite [8]. Vitamin D not only induces epigenomic modifications, but its own metabolism is also subject to epigenetic regulation [11].

## 4. Molecular Basis of Vitamin D Signaling

### 4.1. Vitamin D Metabolism

Vitamin D occurs in two forms: cholecalciferol (vitamin D_3_) and ergocalciferol (vitamin D_2_). It is obtained both endogenously through cutaneous synthesis and exogenously from dietary sources. Endogenous production represents the major source of vitamin D; however, it is strongly dependent on sun exposure and may be limited under conditions of insufficient ultraviolet B (UVB) radiation. Dietary sources of vitamin D_3_ include fatty fish, fish liver oils, and egg yolk, whereas vitamin D_2_ is found mainly in mushrooms. In addition, fortified foods, such as milk, plant-based beverages, and margarine contribute significantly to dietary vitamin D intake [28]. Reduced sunlight exposure due to lifestyle changes and seasonal variations increases the importance of dietary intake and supplementation to maintain adequate 25(OH)D levels [10]. In human skin, cholecalciferol is synthesized from 7-dehydrocholesterol upon exposure to UVB radiation. Both cholecalciferol and ergocalciferol subsequently undergo a series of metabolic transformations to generate biologically active metabolites. The first step, 25-hydroxylation by CYP2R1 and CYP27A1, occurs predominantly in the liver. Notably, 25(OH)D is the major circulating form of vitamin D and is routinely used as a clinical marker of vitamin D status. This is followed by 1α-hydroxylation of 25(OH)D, catalyzed by CYP27B1, primarily in the kidneys, resulting in the formation of the active hormone 1,25(OH)_2_D [29]. A fact worth noting is the expression of *CYP27B1* in extra-renal tissues, including epithelial cells in various organs, the immune, skeletal, and endocrine systems, placenta, brain, liver, and also in tumor cells. However, the extent to which this expression contributes to 1,25(OH)_2_D production, as well as its physiological and clinical significance, remains unclear. It has been postulated that extra-renal 1,25(OH)_2_D primarily exerts local paracrine or autocrine effects [30]. Vitamin D metabolites are transported in the circulation predominantly bound to vitamin D-binding protein (DBP) and, to a lesser extent, albumin. CYP24A1 initiates the catabolic inactivation of 1,25(OH)_2_D, thereby regulating vitamin D homeostasis [29].

### 4.2. Vitamin D Receptor (VDR)

The biological effects of 1,25(OH)_2_D, including its epigenetic actions, are predominantly mediated through binding to VDR [31]. VDR is a member of the nuclear receptor superfamily, whose proteins bind small hydrophobic ligands and function as ligand-activated transcription factors capable of modulating RNA polymerase II activity and gene expression [31,32].

VDR most commonly forms heterodimers with the retinoid X receptor (RXR), although it can also form homodimers or interact with other nuclear receptors, such as the retinoid acid receptor or the thyroid hormone receptor [3]. The VDR/RXR complex preferentially binds to vitamin D response elements (VDREs) [33]. The classical VDRE is a direct repeat with a three-nucleotide spacer (DR3), consisting of two hexameric sequences separated by three nucleotides. However, only approximately 11.5% of VDR binding sites contain this canonical motif, indicating that additional mechanisms contribute to VDR recruitment to chromatin [34]. Beyond canonical VDRE binding, VDR can associate with genomic DNA through cooperation with pioneer transcription factors, including PU.1, CEBPα, GABPA, ETS1, RUNX2, and BACH2, enabling binding at non-VDRE loci [8,10,35,36].

VDR is capable of binding DNA in the absence of ligand; however, activation by 1,25(OH)_2_D enhances heterodimer formation with RXR and promotes high-affinity binding to VDREs [37,38]. In vitro studies have demonstrated a 2- to 10-fold increase in VDR DNA binding following 1,25(OH)_2_D stimulation [36]. The most pronounced increase occurs at DR3-containing sites, which are predominantly located distal to transcription start sites (TSSs). The presence and number of these motifs correlate with stronger and more stable VDR binding [39,40].

VDR is widely expressed across the human body and has been detected in more than half of all human tissues and cell types. Genome-wide analyses reveal a highly cell type-specific VDR cistrome, characterized by thousands of genomic binding sites, with overlap between tissues limited to a small subset of key regulatory regions [3,9]. This cell-specific binding landscape largely explains the cell-dependent diversity of vitamin D-responsive genes. Each cell type contains approximately 1000–10,000 VDR binding sites, substantially exceeding the number of primary 1,25(OH)_2_D target genes, which typically ranges from 200 to 1000 per tissue [40].

Only a small fraction of these binding sites are persistent, and even these exhibit dynamic, ligand-dependent changes in VDR occupancy [36,41]. Persistent VDR binding sites function as primary regulatory hubs for vitamin D signaling, whereas transiently occupied sites may participate in secondary or context-dependent gene regulation [9]. To date, more than 11,000 genes have been proposed as potential VDR targets, encompassing pathways involved in metabolism, cell and tissue morphology, cell adhesion, differentiation and development, angiogenesis, and epithelial-to-mesenchymal transition [33].

Approximately 87% of VDR binding sites are located within regions of open chromatin, which become further activated upon ligand stimulation [3]. Transcriptional activation of primary 1,25(OH)_2_D target genes requires that both the TSS and at least one VDR binding site reside within the same chromatin domain and be embedded in accessible chromatin regions [21]. In contrast, secondary vitamin D target genes are regulated indirectly through transcription factors encoded by primary VDR-responsive genes [42].

Importantly, the VDR gene itself undergoes epigenomic autoregulation. Binding of ligand-activated VDR to VDREs within enhancer regions of the VDR gene locus mediates this feedback mechanism. Autoregulation is thought to involve ligand-dependent chromatin remodeling and coordinated interactions with co-repressors and co-activators, underscoring the central role of VDR in 1,25(OH)_2_D-dependent epigenetic regulation [33].

## 5. Epigenetic Effects of Vitamin D Action

### 5.1. Histone Modifications

#### 5.1.1. Expression of Histone-Modifying Enzymes

1,25(OH)_2_D modulates the epigenome both through direct interactions of the VDR with chromatin-modifying enzymes and via regulation of genes encoding these enzymes [8]. However, the evidence is largely limited to cell culture and animal studies, with few human studies available to translate these mechanistic findings into meaningful biological outcomes.

Stimulation of SW480-ADH human colon cancer cells with 1,25(OH)_2_D increases expression of the histone H3 lysine demethylase *KDM6B/JMJD3*, which partially mediates the transcriptional activation of several vitamin D target genes, including *CYP24A1*, the tumor suppressor *CDH1*/E-cadherin, and *CST5*/cystatin D. The potential role of JMJD3 in mediating the protective effects of 1,25(OH)_2_D in colon cancer is suggested by experimental studies showing that *JMJD3* depletion attenuates key 1,25(OH)_2_D-mediated responses, as well as by observations of reduced *JMJD3* expression in a substantial proportion of colon cancer patients [43]. This effect may be mediated by decreased expression of miR-200b and miR-200c. In the same cell line, 1,25(OH)_2_D was also found to increase expression of other lysine-specific demethylases, including LSD1, LSD2, JARID2, and KDM5B, while suppressing several histone demethylases, such as KDM4A/C/D, KDM5A, KDM2B, JMJD5/6, and PLA2G4B [20].

Ligand-bound VDR exhibits cyclical association with VDRE regions within the insulin-like growth factor binding protein 3 (*IGFBP3*) gene, coinciding with histone H4 deacetylation at these sites. 1,25(OH)_2_D upregulates the histone deacetylases HDAC4 and HDAC6, as demonstrated by combined silencing of HDAC4 and HDAC6, which abolishes this cyclical recruitment [44].

In a rat model of experimental autoimmune encephalomyelitis (EAE), cholecalciferol supplementation was associated with downregulation of several histone-modifying enzymes generally linked to gene repression, including HDAC1, HDAC2, KDM1B, KDM2A, and KDM5A in CD4^+^ T lymphocytes. Together with other epigenetic changes, this effect reduced Th1/Th17 cell proliferation and activity and influenced the expression of multiple sclerosis-associated genes [45].

#### 5.1.2. Recruitment of Co-Modifying Proteins by VDR

The VDR/RXR complex interacts via the outer surface of its ligand-binding domain with nuclear proteins, including co-repressors and co-activators, forming larger regulatory complexes [10]. The composition of these complexes depends on ligand binding and gene context. Unliganded VDR can recruit co-repressor proteins, leading to transcriptional repression. Ligand binding shifts the balance toward co-activator recruitment, promoting gene transcription [46].

Known co-repressors include NCoR1, SMRT/NCoR2 [20], ALIEN/COPS2 [8,47], SIN3A, LCOR [48], DREAM, and HR [49], which exert HDAC activity or associate with HDACs. Co-activators include histone acetyltransferases (HATs) from the SRC family (46), CBP/p300 [49], NCOA family members, Mediator complex proteins [8], ARA54, SKIP, RBP2, SRB7 [48], and CCND3 [50]. Some interacting proteins can act as either co-activators or co-repressors depending on context [48].

In the THP-1 human monocyte cell line, 1,25(OH)_2_D stimulation significantly affected 550 of 22,998 H3K4me3-marked genomic regions (primarily promoters) and 2473 of 45,578 H3K27ac-marked regions (primarily enhancers), identifying 59 vitamin D target genes. These findings suggest that 1,25(OH)_2_D may influence the human epigenome at the level of histone modifications [51]. Experimental treatment with HDAC inhibitors in THP-1 cells suggests that HAT recruitment and H3K27 acetylation are key mechanisms by which 1,25(OH)_2_D alters chromatin structure [46]. In murine mesenchymal stem cells (MSCs), 1,25(OH)_2_D induced enrichment of H3K9Ac at vitamin D-regulated genes, marking active chromatin. Notably, VDR binding and histone modifications at the Mmp13 locus occurred outside of the transcription start site [52]. VDR binding at proximal elements of bone homeostasis and vitamin D metabolism genes (*Spp1*, *Cyp24a1*, *Vdr*, *Tnfsf11*) led to increased H3 and H4 acetylation. Subsequent studies in osteoblasts and osteocytes confirmed gene-specific acetylation of H3K9, H4K5, and H3K27, likely via VDR-mediated recruitment of HATs [53]. In the MCF-12A mammary epithelial cell line, unliganded VDR recruits NCoR1 and HDACs, repressing cyclin-dependent kinase inhibitor (*CDKI*) genes. Both HDAC inhibitors and 1,25(OH)_2_D suppress cell proliferation via CDKI induction, but target different CDKI families (INK4 vs. Cip/Kip) [54].

### 5.2. DNA Methylation

#### 5.2.1. Global Methylation Changes

1,25(OH)_2_D appears to modulate global DNA methylation in cellular and animal models by regulating the expression of DNA methyltransferases (DNMTs) and DNA demethylases. In dendritic cells, VDR promotes demethylation of its binding sites through interaction with TET2 [55]. In animal models, CD4^+^ T cells from cholecalciferol-supplemented animals showed reduced expression of *DNMT1*, *DNMT3a*, and *DNMT3b* [45]. Similarly to retinoid acid, 1,25(OH)_2_D-induced DNA methylation changes in cancer cells may involve negative regulation of DNMT1 via p21 upregulation and AP-1 inhibition. Activation of PTEN, another AP-1 inhibitor, may further downregulate AP-1-responsive genes, including DNMT1 [56]. 1,25(OH)_2_D has also been proposed to prevent hypermethylation of promoter regions of multiple diabetes-related genes by increasing DNA demethylase expression [57]. However, in peripheral blood mononuclear cells (PBMCs), substantial genome-wide methylation changes have not been observed after 1,25(OH)_2_D in vitro stimulation [58].

LINE-1 methylation, a surrogate marker of global DNA methylation and genomic stability, has been studied in relation to vitamin D exposure. In South Australian participants, personal solar UV exposure correlated negatively with LINE-1 methylation; however, serum 25(OH)D levels were not associated with DNA methylation and did not modify the UV–methylation relationship [59].

One-year supplementation with cholecalciferol, calcium, and B vitamins did not alter LINE-1 methylation in blood cells [60]. Cross-sectional studies in elderly Swedish populations found no association between total vitamin D intake (diet, supplements, sun exposure) and global methylation; however, supplementation, including vitamin D, was linked to differential methylation in the *SLC25A24* gene [61]. In African Americans, plasma 25(OH)D positively correlated with global 5-methylcytosine (%5-mC), and a 16-week cholecalciferol supplementation trial increased global DNA methylation in a dose-dependent manner in vitamin D-deficient individuals, an effect not observed in Caucasians [62].

Evidence for the effects of 1,25(OH)_2_D on methylation patterns is currently derived mainly from cell culture and animal studies and has not yet been consistently confirmed in observational studies or randomized controlled trials. In human studies, the majority of which were observational studies, either no significant changes have been observed, or the reported effects appear to be site-specific and dependent on baseline 25(OH)D status or ethnicity [59,60,61,62].

#### 5.2.2. DNA Methylation Age

DNA methylation age (DNAm age) and DNA methylation age acceleration (DNAmAA) estimate biological age based on DNA methylation patterns relative to chronological age. Various epigenetic clocks are used to assess DNAm age and DNAmAA, with established associations to morbidity and mortality [63].

In the population-based Berlin Aging Study II (BASE-II), sufficient serum 25(OH)D concentrations were associated with lower mean DNAmAA (7-CpG clock) and longer relative leukocyte telomere length. However, neither parameter was significantly associated with the Fried frailty phenotype or functional assessments [64]. In contrast, another population-based study involving a larger cohort of older adults reported no significant associations between serum 25(OH)D levels and either leukocyte telomere length or epigenetic age acceleration assessed using Horvath’s and Hannum’s clocks [65].

A subsequent quasi-interventional analysis conducted within the BASE-II elderly sub-cohort produced similarly inconclusive findings. Self-administered cholecalciferol supplementation was associated with significantly lower DNA methylation age estimates according to two of five epigenetic clocks evaluated (7-CpG and Horvath’s clocks) when compared with untreated vitamin D-deficient participants. Due to the study design, causal inference cannot be established [63].

In a study of African American adults with overweight or obesity (*n* = 51), cholecalciferol supplementation, as well as increases in serum 25(OH)D concentrations independent of supplementation, were associated with reductions in epigenetic age estimated by Horvath’s clock, whereas no significant changes were observed using Hannum’s clock [66].

Evidence from interventional studies remains limited, and most available studies are non-randomized or uncontrolled. A one-year non-controlled pilot trial involving 120 participants aged 65–79 years from the NU-AGE cohort did not demonstrate a significant overall effect of a Mediterranean diet supplemented with 400 IU cholecalciferol on DNAm age assessed by Horvath’s clock. However, a significant reduction in epigenetic age was observed among Polish women and participants with higher baseline epigenetic age [67]. Additionally, deceleration of selected epigenetic clocks, including PhenoAge and GrimAge, has been reported in patients with mild cognitive impairment receiving cholecalciferol and/or marine omega-3 supplementation [68].

Overall, current evidence regarding the association between serum 25(OH)D levels or cholecalciferol supplementation and epigenetic aging remains inconsistent. This inconsistency likely reflects the limited number of available studies, heterogeneity in study populations, differences in supplementation regimens and duration, and the use of diverse epigenetic clocks. Furthermore, the predominance of observational, non-controlled, and non-randomized study designs limits the ability to establish causality and constrains potential clinical applications.

#### 5.2.3. Influence of Maternal 25(OH)D Level on Offspring DNA Methylation

Maternal 25(OH)D is the sole source for the fetus [18], and deficiency is linked to adverse pregnancy outcomes, including gestational diabetes mellitus, preterm labor, low birth weight, and preeclampsia [69]. Maternal diet during pregnancy may influence offspring phenotype via epigenetic mechanisms [19].

Animal studies show gestational vitamin D deficiency induces DNA methylation changes at imprinted loci across generations, affecting body weight in a cell type- and generation-specific manner. Genome-wide analyses in mice revealed decreased methylation in offspring with higher body and testes weight, involving genes in cadherin, Wnt, PDGF, integrin signaling, and angiogenesis [70]. Vitamin D deficiency in mothers has been associated with offspring obesity (*Vldlr*, *Hif1α*), persistent inflammation and insulin resistance (*IκBα*), altered osteoblast differentiation (*Osterix*, *Runx2*), and elevated blood pressure (*Pannexin-1*) [71,72,73,74].

**Table 1:** Effects of maternal vitamin D deficiency on offspring methylation patterns: evidence from animal studies.

Study Subject	Intervention/Exposure	Epigenetic Effect	Phenotypic Effect	Overall Conclusions
Collaborative Cross (CC) inbred mice— two generations	Maternal vitamin D deficiency before and during pregnancy	Altered DNA methylation at imprinted loci (e.g., *H19/Igf2*, *Snrpn*, *Dlk1/Gtl2*); mostly hypomethylation in liver and sperm	Changes in body weight across two generations (increase in F1, decrease in F2); effects dependent on parent of origin	Maternal vitamin D deficiency leads to heritable epigenetic changes in both germline and soma, affecting the phenotype of subsequent generations [70]
Wistar rat offspring	Maternal vitamin D deficiency	Differential methylation of 608 promoters and 204 CpG islands in vitamin D-deficient mothers’ offspring	Increased body weight, fat tissue volume, glucose levels, and blood lipids; enhanced pre-adipocyte proliferation	Maternal vitamin D deficiency promotes adipogenesis and fat accumulation in offspring through epigenetic modifications [71]
Sprague–Dawley rats and their offspring	Maternal vitamin D deficiency	↑ *IκBα* methylation in vitamin D-deficient mothers’ offspring	Persistent inflammation (↑ IL-1β, IL-6 IL-8, and TNF-α), impaired glucose metabolism, and insulin resistance	Increased inflammation and impaired glucose metabolism due to maternal vitamin D deficiency possibly modulated by epigenetic mechanism [72]
Sprague–Dawley rat offspring	Maternal vitamin D deficiency	Hypermethylation of the *Panx1* gene promoter in offspring	Increased arterial blood pressure and impaired vascular function (endothelium-dependent relaxation)	Maternal vitamin D deficiency increases the risk of hypertension in offspring via Panx1 hypermethylation [74]
Mice offspring (analysis of tibiae)	Vitamin D-replete vs. vitamin D-depleted maternal diet with bone mechanical loading in offspring	Altered methylation of *Osterix* and *Runx2* promoters (due to vitamin D deficiency); reduced *Rxra* methylation (due to loading)	Reduced bone mass and impaired bone accrual; bone strength correlated with methylation of *Vdr*, *Osterix*, and *Rxra*	Both diet and mechanical factors modulate the methylation of key bone genes, determining skeletal development and strength [73]

↑ increase/upregulation.

Clinical studies, however, have yielded inconclusive results. Population-based studies have linked maternal vitamin D deficiency with adverse clinical outcomes, but these effects have not been consistently shown to be mediated by methylation changes. Moreover, the observed methylation patterns appeared to be CpG site-specific and potentially influenced by ancestry and environmental factors. Although the involvement of *RXRA* methylation has been suggested, findings from observational studies and randomized controlled trials, including clinical endpoints, remain inconsistent.

Population-based studies (PACE consortium) generally found no association between maternal 25(OH)D levels and global or gene-specific methylation in cord blood [75]. In neonatal cord blood, *CpG* methylation was influenced by ancestry and maternal 25(OH)D serum level, but no single gene was exclusively associated with 25(OH)D levels [76]. Lower maternal 25(OH)D was associated with lower birth weight and higher childhood obesity risk, but specific methylation changes in growth-related genes were not detected [77].

In an RCT, prenatal and postnatal cholecalciferol supplementation altered leukocyte methylation in mothers and infants, affecting genes involved in cell migration, cadherin signaling, collagen metabolism, transcription, immune function, and apoptosis [78].

Epigenomic regulation of retinoid-X-receptor-alpha (RXRA) is suggested to play a critical role in bone health. *RXRA* hypermethylation has been linked to preeclampsia [79]. In vitro treatment of six human placentas with 25(OH)D modulated *RXRA* promoter methylation in a CpG site-dependent manner, resulting in an overall increase in *RXRA* expression [80]. Observational data from the Southampton Women’s Survey (SWS) indicated that maternal free 25(OH)-vitamin D index was negatively associated with *RXRA* methylation in offspring DNA. Reduced methylation—and consequently, higher *RXRA* expression—correlated with increased bone mineral content (BMC) at 4 years of age [81]. Conversely, the Maternal Vitamin D Osteoporosis Study (MAVIDOS) randomized controlled trial reported no effect of gestational cholecalciferol supplementation on neonatal whole-body BMC [82]. Post hoc analysis, however, revealed a general decrease in *RXRA* promoter methylation in offspring of supplemented mothers, particularly during summer, though statistical significance was observed in only one of three CpG clusters. Notably, maternal serum 25(OH)D levels did not consistently associate with *RXRA* methylation in the umbilical cord, and *RXRA* methylation correlated with neonatal bone indices in the placebo but not the supplementation group [80]. These findings highlight mechanistic links between maternal 25(OH)D status, *RXRA* methylation, gene expression, and offspring bone development. Inconsistencies regarding *RXRA* methylation effects on children’s BMC may reflect differences in age at measurement (newborns vs. 4-year-olds), suggesting the influence of additional environmental factors [73,80,82]. RXRA’s broad interactions with other nuclear receptors—including VDR, thyroid hormone receptor, glucocorticoid receptor, and PPARs—may further modulate bone mass outcomes [80,81].

Compared with DNA methylation age, gestational DNA methylation age (DNAmGA) remains less studied, and its clinical relevance for neonatal outcomes is limited. DNAmGA acceleration has been linked both to adverse maternal conditions and to favorable pregnancy outcomes. Post hoc analyses of RCT data indicated that higher-dose maternal cholecalciferol supplementation was associated with DNAmGA deceleration in African American participants, potentially reflecting lower baseline 25(OH)D serum level [83]. Similarly, population-based studies reported lower epigenetic age acceleration in offspring of mothers who used vitamin D supplements during pregnancy [84].

#### 5.2.4. Cancer

Carcinogenesis is driven by both genetic mutations and epigenetic alterations. Epigenetic changes promoting the initiation and progression of cancer include global DNA hypomethylation, which contributes to genomic instability, and locus-specific promoter hypermethylation, leading to silencing of tumor suppressor genes [85].

##### Colorectal Cancer (CRC)

In colorectal cancer (CRC) patients, low serum 25(OH)D levels were associated with global DNA hypomethylation in tumor tissue [86]. In healthy subjects, 25(OH)D serum level was negatively associated with methylation of tumor suppressor gene promoters (APS) and slightly positively correlated with LINE-1 methylation in rectal mucosal cells [87]. As mentioned, LINE-1 methylation serves as an index of global DNA methylation, and its hypomethylation is linked to genomic instability [85,87]. CRC patients displayed reduced LINE-1 methylation in both tumor tissue and visceral adipose tissue compared with controls, with methylation levels positively associated with 25(OH)D, particularly among individuals with obesity. Logistic regression indicated that higher LINE-1 methylation was associated with lower CRC risk, whereas serum 25(OH)D levels did not show a significant effect [88]. In visceral adipose tissue of CRC patients, *DNMT3A* expression was inversely correlated with 25(OH)D and positively associated with *VDR* promoter methylation, suggesting a potential epigenetic regulatory mechanism [89].

In humans, observational data linked overall vitamin D intake (assessed by food frequency questionnaire, including dietary sources and supplements) with reduced promoter methylation of *DKK1*, a canonical Wnt pathway inhibitor with anti-proliferative and pro-apoptotic effects that is frequently silenced in CRC [90]. Conversely, hypermethylation of *SFRP2* promoters leads to constitutive Wnt pathway activation in CRC. Patients with higher 25(OH)D levels exhibited decreased *SFRP2* promoter methylation in tumor cells, but not in PBMCs or visceral adipose tissue. However, in vitro treatment of HCT116 colorectal carcinoma cells with cholecalciferol did not affect *SFRP2* methylation [91]. The inconsistency between in vitro experimental exposure and observational human studies may be explained by several factors, including longer-term exposure in humans, the absence of the active form of vitamin D in cell culture experiment, and a more complex, potentially indirect relationship between *SFRP2* methylation and circulating 25(OH)D levels. Alternatively, the observed associations may be correlative rather than causal and influenced by additional confounding factors. Treatment with 1,25(OH)_2_D induced *DKK1* expression in CRC cells, accompanied by demethylation of CpG sites in its promoter region [92].

##### Breast Cancer

In breast cancer cell lines, 1,25(OH)_2_D treatment induced demethylation of promoters of *CDH1* with a concurrent increase in gene expression [93]. In a case-cohort study of breast cancer patients, serum 25(OH)D concentrations were associated with DNA methylation of several vitamin D-related genes, including *RXRA*, *CYP24A1*, *CYP27B1*, *NADSYN1/DHCR7*, and *VDR*, suggesting potential modulation of breast cancer risk via epigenetic mechanisms [94].

#### 5.2.5. Methylation as a Regulatory Mechanism of Vitamin D Metabolism

Genome-wide observational studies suggest that DNA methylation of genes involved in vitamin D metabolism may be associated with the effect of vitamin D supplementation on 25(OH)D serum level. It is suggested that genetic variation can only predict 20% of the variation in vitamin D response indices, while the remaining could be due to epigenetic variations. According to the studies, the methylation status of *CYP2R1*, *CYP27B1*, *CYP24A1*, and *VDR* genes is responsible for nearly 18% of the 25(OH)D level variance [95]. This epigenetic variation might be a contributing factor to vitamin D deficiency [96].

In an elderly Swedish cohort, methylation status of the 80 vitamin D-related genes did not change with vitamin D supplementation [61]. In a small cohort of African American adolescents (*n* = 22), small differences in DNA methylation of *CYP2R1* and *CYP24A1* were observed between groups stratified by serum 25(OH)D status. Specifically, higher methylation of *CYP2R1* and lower methylation of *CYP24A1* were detected in individuals with vitamin D deficiency. However, due to the observational nature of the study, causal relationships between methylation changes and serum 25(OH)D status could not be established [97]. An observational study in elderly Australians found a weak negative correlation between serum 25(OH)D and *CYP2R1* methylation, and a positive correlation with *VDR* methylation, suggesting a potential feedback mechanism where higher 25(OH)D serum level reduces receptor expression to maintain homeostasis. The positive association between *CYP24A1* methylation and 25(OH)D was attenuated after adjusting for vitamin D intake. No association was observed between 25(OH)D and *CYP27B1* methylation. Vitamin D intake was independently positively associated with methylation of *CYP2R1* and *CYP24A1* [98]. In a study in patients with colorectal cancer, serum 25(OH)D was positively associated with methylation and mRNA expression of VDR and CYP27B1 genes in visceral adipose tissue [99]. A weak association between higher methylation of the *CYP27B1* gene and lower 25(OH)D serum level and none with *CYP24A1* was shown in a population-based study in PBLs [100]. In one case–control study on 122 patients with pulmonary tuberculosis and 118 healthy controls, the methylation of a fragment (cumulative methylation of CpG sites at the specific region of the gene) at the *CYP27A1* showed a positive correlation with 25(OH)2D level [101]. Further insights were provided by a post hoc analysis of the Calcium and Vitamin D Malnutrition in Elderly Women Study (CaMEWS). Participants with the most extreme changes in serum 25(OH)D following supplementation (*n* = 36) were classified as responders or non-responders. In both groups, cholecalciferol supplementation was associated with decreased methylation of *CYP24A1*, while no significant changes were detected in *CYP2R1*, *CYP27A1*, or *CYP27B1*. However, a validation study conducted in the entire cohort, without stratification into responders and non-responders and focusing on individual CpG sites, demonstrated that cholecalciferol supplementation decreased methylation of *CYP2R1* and influenced *CYP24A1* methylation in a CpG site-specific manner [102].

Overall, these studies are limited by small sample sizes and, with the exception of the CaMEWS cohort, predominantly observational designs. Importantly, only one of the studies assessed the functional consequences of methylation changes by measuring gene expression at the mRNA, while none assessed protein level. An additional limitation is that DNA methylation, undoubtedly for practical reasons, was assessed in DNA circulating in serum, leukocytes, and peripheral blood cells (PBCs) rather than in primary target organs of vitamin D metabolism, such as the liver and kidneys. Nevertheless, the available evidence suggests that cholecalciferol supplementation can modulate DNA methylation of genes involved in its metabolism in a gene- and CpG site-specific manner. Methylation differences appear to be more pronounced in individuals with extreme serum 25(OH)D concentrations or responses to supplementation. Notably, the direction of the association between 25(OH)D status and DNA methylation of *CYP24A1* is not fully consistent across studies. Beckett et al. suggest that increased methylation of *CYP24A1* in response to increased 25(OH)2D serum level may represent a compensatory mechanism contributing to the maintenance of vitamin D homeostasis rather then decreased methylation being a cause of vitamin D deficiency [98].

### 5.3. Non-Coding RNAs

1,25(OH)_2_D regulates miRNA expression primarily through VDR-mediated binding to VDREs in the proximity of miRNA genes, with evidence for negative feedback loops between certain miRNAs and VDR [10,24,45]. Animal studies have shown that cholecalciferol supplementation increases expression of snRNA, miRNA, and ribosomal RNA genes [45], but a 12-month RCT in humans (40,000 IU/week) did not detect consistent changes in circulating miRNAs, suggesting systemic effects may be subtle, tissue-specific, or context-dependent [103].

#### 5.3.1. Metabolic Functions

In endothelial progenitor cells exposed to high glucose, 1,25(OH)_2_D altered the expression of hundreds of mRNAs, lncRNAs, circRNAs, and miRNAs, improving cellular function via ceRNA networks [104]. In diet-induced obese mice, a cholecalciferol-supplemented diet reduced expression of pro-inflammatory miRNAs (miR-146a, miR-150, miR-155) in adipose tissue, likely via NF-κB inhibition [105], and altered miRNA content in adipocyte-derived extracellular vesicles [106]. High-dose cholecalciferol supplementation in rats downregulated hepatic miR-122 and miR-9, associated with insulin resistance and inflammation, though metabolic improvements were also observed at moderate-dose supplementation without miRNA changes [107]. In type 2 diabetes animal models, cholecalciferol supplementation alone or combined with aerobic exercise improved glucose metabolism, reduced body weight, and modulated cardiac miRNAs (miR-1) and growth factors (IGF-1, VEGF-B) [108,109]. In metabolic dysfunction-associated steatotic liver disease (MASLD) models, cholecalciferol supplementation alleviated metabolic and inflammatory alterations via miR-155, miR-33a, and miR-200c modulation [110,111].

Evidence from animal models appears to translate to humans. In MASLD patients, 12-week cholecalciferol supplementation (4000 IU/day) improved liver and lipid markers and downregulated profibrogenic miR-21 and antifibrogenic miR-122 [112]. In prediabetic individuals, cholecalciferol supplementation 2000 IU/day for 4 months reduced miR-7 and miR-192 and increased miR-152, correlating with HbA1c and fasting glucose changes [113].

#### 5.3.2. Cancer

**Figure 1:**
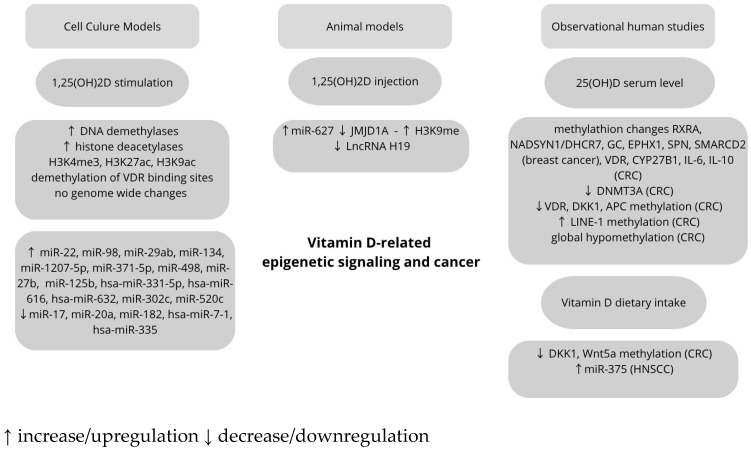
Vitamin D-related epigenetic signaling and cancer.

Human tumors are characterized by a widespread reduction in global miRNA expression. Specific miRNAs have been implicated in cancer initiation and progression [24]. An overview of epigenetic mechanisms linked to vitamin D and cancer is shown in Figure 1.

In SW480 colon cancer cells, 1,25(OH)_2_D upregulated miR-22, suppressing OGN, NELL2, and NFAT5, mediating antiproliferative and anti-migratory effects [114]. Consistently, miR-22 levels were reduced in patient tumor samples and positively correlated with *VDR* expression. In HT-29 cells, 1,25(OH)_2_D induced miR-627, which suppressed the histone demethylase JMJD1A, increased H3K9 methylation, and reduced expression of proliferative factors, ultimately inhibiting cell proliferation and xenograft growth in an animal model. miR-627 was also lower in patient tumor samples [115]. Additionally, VDR downregulated the long non-coding RNA H19 via c-Myc/Mad-1 in cell models, while H19 reduced VDR expression through miR-675-5p, suggesting a mechanism of resistance to 1,25(OH)_2_D [116].

1,25(OH)_2_D stimulation increased miR-100 and miR-125b in a prostate cancer cell line (LNCaP) and in animal models, downregulating oncogenes *PLK1* and *E2F3* and reducing proliferation, migration, and invasiveness [117]. Combined 1,25(OH)_2_D and testosterone in cell cultures altered 15 miRNAs, including miR-22, miR-29a/b, miR-134, and miR-98, targeting cell cycle and metabolic genes [24,118]. Human studies, including pre-prostatectomy cholecalciferol supplementation, demonstrated increased intraprostatic 1,25(OH)_2_D levels and upregulation of miR-100, miR-125b, and piRNAs in benign and malignant epithelial cells [117,119].

In breast cells lines (MCF12F and MCF-7), 25(OH)D/1,25(OH)_2_D modulated miR-26b, miR-182, miR-18a, miR-106, miR-30c, and miR-125b, contributing to stress protection and antitumor effects [120]. In MCF-7 breast cancer cells, miR-125b was shown to downregulate *VDR* expression, and its reduced expression in tumors may amplify the antitumor effects of 1,25(OH)_2_D [121,122]. In ovarian cancer cells, 1,25(OH)_2_D upregulated miR-498 via VDR/RXR, suppressing *hTERT* and reducing tumor growth [123]. 1,25(OH)_2_D and the cholecalciferol synthetic analogue tacalcitol increased miR-27b and miR-125b in leukemia and lymphoma cell lines, mediating antiproliferative effects [124]. Notably, miR-125b has also been implicated in early melanoma progression and metastasis, suggesting a broader role for 1,25(OH)_2_D-regulated miRNAs in tumor biology [24].

Vitamin D intake was associated with increased miR-375 expression in patients in head and neck squamous carcinoma [125]. In CAL-27 and SCC-25 cell lines, cholecalciferol modulated miR-7-1-3p, miR-335, miR-331-5p, miR-616-3, and miR-632, affecting proliferation, angiogenesis, and MAPK/WNT signaling [126].

In keratinocytes and epidermal tissue, VDR deficiency increased oncogenic lncRNAs (H19, HOTTIP, Nespas) and suppressed tumor-suppressive lncRNAs (Kcnq1ot1, lincRNA-p21), highlighting a mechanistic role for vitamin D signaling in protecting against skin cancer development [127].

In breast cancer and acute myeloid leukemia models, 1,25(OH)_2_D enhanced NK cell cytotoxicity via miR-302c and miR-520c downregulation, suggesting immunomodulatory antitumor mechanisms [128].

#### 5.3.3. Immune Response

1,25(OH)_2_D modulates innate immunity through miRNAs. In monocyte-derived macrophages, 1,25(OH)_2_D treatment downregulated miR-6501-3p, miR-1273h-5p, miR-665, miR-1972, miR-1183, and miR-619-5p, regulating PDCD1LG2, IL1B, and CD274 [129]. In DENV-2-infected monocyte-derived macrophages, 1,25(OH)_2_D stimulation reduced miR-182-5p, miR-130a-3p, and miR-125b-5p, increasing IFN-I, OAS1, and SOCS1 without affecting viral replication [130]. In porcine rotavirus models, dietary supplementation with cholecalciferol suppressed miR-155-5p, restoring TBK1/IRF3 signaling and interferon-β production [131].

Evidence from human studies further supports the immunomodulatory role of 1,25(OH)_2_D-regulated miRNAs. Ultramarathon runners receiving 10,000 IU/day cholecalciferol showed increased TNF-α and miR-155 and reduced IL-1β compared with placebo [132]. In multiple sclerosis patients, cholecalciferol supplementation reduced miR-155-5p expression [133]. miR-155 plays a tissue-context-dependent role—in skeletal muscle, it is postulated to play a role in regeneration processes, whereas in the immune system its activity is strongly pro-inflammatory. In inflammatory bowel disease patients, cholecalciferol supplementation increased miR-150-5p, Let-7a-5p, miR-30d-5p, and Let-7f-5p in circulating extracellular vesicles [134].

#### 5.3.4. Respiratory System

1,25(OH)_2_D mitigates airway inflammation via miR-21-5p, miR-27b, miR-145-5p, miR-146a-5p, and miR-155-5p modulation. In human bronchial fibroblasts, 1,25(OH)_2_D reduced miR-21-5p, inhibiting TGF-β/Smad signaling and fibrotic remodeling [135]. 1,25(OH)_2_D downregulated miR-27b in human lung fibroblasts, affecting fibrosis [136]. Porcine models showed that oral supplementation of cholecalciferol and calcidiol altered lung miRNAs (miR-150, miR-193, miR-145, miR-574, miR-340, miR-381, miR-148, miR-96) linked to inflammation, asthma, and immune pathways [137].

#### 5.3.5. Other

In human placental tissue, 1,25(OH)_2_D upregulated miR-181b-5p and miR-26b-5p, inhibiting CRH and COX-2, which may be a molecular mechanism through which 1,25(OH)_2_D may exert protective effects against preterm labor [138].

In osteoblasts, 1,25(OH)_2_D modulated miR-1228 and miR-637, targeting *BMP2K* and *COL4A1* [139].

Short-term supplementation with cholecalciferol, olive oil, and omega-3 fatty acids altered three tRNAs, 15 miRNAs, and 112 piRNAs involved in fatty acid metabolism and transposable elements [140].

In 25(OH)D-deficient patients undergoing coronary artery bypass, miR-21 expression was elevated in aorta and mammary arteries, though correcting 25(OH)D serum level did not alter circulating miR-21 despite cardiovascular improvements [141].

### 5.4. Three-Dimensional Chromatin Structure

Most VDR binding sites are located within open chromatin regions, with a marked increase in chromatin accessibility following stimulation with 1,25(OH)_2_D [40]. This chromatin-opening effect of 1,25(OH)_2_D appears to be more pronounced under conditions of immune activation. In THP-1 cells, co-stimulation with 1,25(OH)_2_D and lipopolysaccharide (LPS), mimicking bacterial infection, induces substantially greater changes in chromatin accessibility than either treatment alone [142]. Comparison of the THP-1 cellular model and in vitro PBMC studies (VitDmet, VitDbol) demonstrated substantial overlap in affected genomic loci, with approximately 85% shared regions, as well as similar patterns of chromatin accessibility changes following cholecalciferol supplementation [143].

Beyond the epigenetic mechanisms described above, higher-order chromatin organization into larger and smaller loops plays a crucial role in chromatin accessibility. Chromatin looping subdivides the genome into chromosomal domains within which genes, their transcription start sites (TSSs), and active enhancer regions are brought into spatial proximity, thereby modulating gene expression [42]. A key regulator of this process is the CCCTC-binding factor (CTCF), which, together with cohesin and other architectural proteins, establishes the boundaries of these domains, known as topologically associating domain (TAD) anchors [8,31].

In THP-1 human monocytes, approximately 44,000 CTCF binding sites have been identified, more than 2000 of which are significantly modulated by 1,25(OH)_2_D stimulation. Notably, about half of these 1,25(OH)_2_D-responsive CTCF sites are involved in chromatin looping and mark TAD boundaries that contain both VDR binding sites and vitamin D target genes. This mechanism may account for up to 70% of vitamin D-dependent gene regulation. Several mechanisms have been proposed to explain how 1,25(OH)_2_D influences CTCF binding, including co-localization of VDR and CTCF binding sites, preferential localization of CTCF within 1,25(OH)_2_D-sensitive chromatin regions, and positioning of 1,25(OH)_2_D-responsive CTCF sites near TSSs of vitamin D target genes, facilitating enhancer–promoter looping. However, additional mechanisms are likely involved and remain to be elucidated [144].

Functionally, CTCF silencing affects approximately 30% of 1,25(OH)_2_D target genes, resulting in either enhanced or suppressed transcription. This effect is thought to arise from altered chromatin architecture that disrupts physical contact between VDR-bound enhancers and the TSSs of target genes [34].

In MDA-MB453 breast cancer cells, 1,25(OH)_2_D induces cyclical transcription factor binding associated with chromatin looping of distal VDREs to the TSS of the *p21* gene and simultaneous transcriptional activation, with observed involvement of histone-modifying enzymes such as HDAC4 and LSD1 [145].

In RWPE1 non-malignant prostate cells, 1,25(OH)_2_D was linked to increased CTCF occupancy within the *KLK* gene cluster, altering chromatin architecture and enabling more selective gene regulation. However, CTCF knockdown does not abolish KLK inducibility by VDR ligands, suggesting additional contributors such as DNA methylation and cohesin (RAD21) in maintaining 3D structure [146].

In human β-like cell culture and murine T2D models, ligand-dependent VDR binding to chromatin remodeling complexes regulates β cell protection. Acetylation of lysine in VDR is recognized by BRD7 or BRD9, which then cooperates with SWI/SNF family chromatin remodeling complexes: PBAF or BAF, respectively. Ligand-induced VDR–PBAF interaction increases chromatin accessibility and enhancer activity, promoting anti-inflammatory gene expression, restoring β cell function, and improving hyperglycemia [147].

SWI/SNF expression levels were reduced in patients with chronic rhinosinusitis. A positive correlation between SWI/SNF protein expression and VDR expression was observed. Stimulation of human nasal epithelial cells (HNECs) with 25(OH)D increased SWI/SNF protein expression, suggesting a potential protective anti-inflammatory role through activation of the chromatin remodeling complex [148].

## 6. 1,25(OH)_2_D as an Epigenetic Modulator—A Review of the Available Evidence

A total of 90 studies were analyzed, highlighting the role of 1,25(OH)_2_D as a factor modulating epigenetic regulation at multiple levels. A detailed summary of the findings is presented in Table 1, Table 2, Table 3, Table 4 and Table 5, which include experimental, observational, population-based, and interventional studies, enabling a comprehensive assessment of both the underlying biological mechanisms and the clinical relevance of vitamin D.

The largest group comprised experimental studies (*n* = 45), including cell culture (*n* = 28), animal models (*n* = 12), and mixed models (*n* = 5). Observational studies accounted for 17 analyses, population-based studies for eight, and interventional studies for 15, including nine randomized controlled trials (RCTs), two post hoc analyses of RCTs, three single-arm prospective studies, and one non-randomized interventional study. Additionally, five of the included studies had a mixed design (preclinical and human study).

A summary of key findings from the analyzed studies is presented in Table 1, Table 2, Table 3, Table 4, Table 5 and Table 6.

Experimental studies conducted in cellular models have demonstrated that vitamin D and its metabolites, particularly 1,25(OH)_2_D, are capable of modulating the expression of more than 30 miRNAs, with the direction of these changes depending on the cell type, the form of vitamin D administered, and the biological context. Table 2 summarizes the effects of vitamin D and its metabolites on miRNAs associated with the regulation of immune and inflammatory responses (miR-146a, miR-155, miR-181b, miR-125b) [105,106,130,131], proliferation and survival of cancer cells (miR-22, miR-98, miR-498, miR-302c, miR-520c) [114,117,118,123,128], regulation of genes involved in the cell cycle, lipid metabolism, and calcium homeostasis (miR-29ab, miR-134, miR-1207-5p, miR-371-5p) [118], fibrosis and tissue remodeling (miR-21-5p, miR-27b) [134,135], as well as bone metabolism (miR-1228, miR-637) [139].

Among the studies presented, the involvement of miR-22 in the anticancer activity of vitamin D [114,118], miR-498 in the inhibition of hTERT telomerase expression [123], and miR-146a, miR-155, miR-21-5p, and miR-27b in the regulation of inflammatory, immune, and fibrotic processes [105,106,130,131,135] has been well documented. However, it should be emphasized that for some miRNAs, particularly miR-181a, miR-181b, miR-125b, and miR-27b, the direction of expression changes differed across experimental models, indicating a strong dependence of the observed effects on cell type and study conditions [24,117,120,121,122,124,130,131,135,136,138].

Table 3 summarizes seven animal-model studies demonstrating that supplementation with vitamin D or its metabolites may modulate the expression of miRNAs and epigenetic enzymes. Reduced expression of HDAC1, HDAC2, KDM1B, KDM2A, KDM5A, DNMT1, DNMT3a, and DNMT3b was observed, accompanied by a global decrease in DNA methylation and increased expression of snRNA, miRNA, and rRNA, which may indicate broad alterations in epigenetic regulation and RNA expression-related processes [45]. These effects were associated with inflammation, lipid metabolism, endothelial function, and immune responses.

With regard to miRNAs, decreased expression of miR-122, potentially associated with liver function and lipid metabolism, as well as miR-9, linked to improved metabolic parameters, was demonstrated [107]. Reduced expression of miR-1 (in the physical activity and vitamin D supplementation group) co-occurred with increased expression of VEGF-B and IGF-1, suggesting a potential role in the regulation of angiogenesis and tissue repair processes [108]. A similar effect was observed for reduced expression of miR-15a and miR-146a, which correlated with increased expression of VEGF, PI3K, and eNOS [109]. Regulation of the immune response was associated with the miR-155/TLR7 axis [110]. In models of hepatic steatosis, increased expression of miR-33a and miR-200c was observed, which may be related to lipid metabolism and liver tissue remodeling [111]. In pulmonary models, supplementation with cholecalciferol and calcidiol altered the expression of several miRNAs, including miR-150, miR-193, miR-145, miR-574, miR-340, miR-381, miR-148, and miR-96; higher doses of cholecalciferol additionally affected miR-215 expression, whereas calcidiol did not exert a significant effect on miRNA expression despite a marked increase in 25(OH)D concentration [137].

These findings suggest that modulation of miRNA expression may represent one of the potential epigenetic mechanisms underlying the in vivo actions of vitamin D, participating in the regulation of metabolic, inflammatory, immune, and vascular processes, alongside alterations in DNA methylation and the activity of epigenetic enzymes.

These findings indicate that the role of 1,25(OH)_2_D as an epigenetic modulator is well supported at the biological level; however, its full translational and clinical significance remains to be established, as the number of clinical studies is still relatively limited.

An analysis of the available data further suggests that, beyond its classical function as a transcriptional regulator via the VDR, 1,25(OH)_2_D may act as an epigenetic modulator, influencing the activity of genes involved in metabolism, immune responses, bone development, fibrotic processes, carcinogenesis, ageing, and fetal programming.

1,25(OH)_2_D may influence three major levels of epigenetic regulation: DNA methylation, histone modifications, and the expression of non-coding RNAs, particularly miRNAs.

Experimental studies indicate that 1,25(OH)_2_D does not act solely as a classical ligand of the VDR but may also participate in the reorganization of the chromatin landscape and regulation of gene expression through changes in chromatin accessibility, modulation of the activity of epigenetic enzymes such as HDACs, DNMTs, and TET2, as well as alterations in histone marks, including H3K27ac, H3K4me3, and H3K9ac [8,20,44,45,46,51,52].

The effect of 1,25(OH)_2_D on miRNA expression was reported particularly frequently, representing one of the most consistently observed findings across the analyzed studies. These observations suggest that modulation of non-coding RNAs may constitute a key epigenetic mechanism underlying the action of 1,25(OH)_2_D, mediating the link between receptor signaling and cellular responses. The most frequently reported miRNAs included miR-155, miR-146a, miR-21, miR-122, miR-22, miR-27b, and miR-182, which are involved in the regulation of inflammatory processes, cell proliferation, metabolism, and, to a lesser extent, fibrosis [105,106,107,109,110,112,114,118,120,124,130,131,132,133,135,136,139].

The strongest biological evidence for 1,25(OH)_2_D action relates to the immune system and inflammatory processes, where numerous studies indicate its immunomodulatory, anti-inflammatory, and antiviral effects. These effects are accompanied by changes in the expression of immune-related genes, cytokines, and miRNAs involved in the regulation of inflammatory responses [45,72,105,106,129,130,131,132,133,137].

Another intensively investigated area is oncology. The available evidence suggests that 1,25(OH)_2_D may exert anti-tumor effects, associated with the inhibition of tumor cell proliferation and migration, suppression of tumor growth, and regulation of tumor suppressor gene expression. These effects have been demonstrated in models of colorectal, prostate, and ovarian cancers, as well as head and neck cancers, leukemias, and lymphomas [43,86,87,88,89,90,92,94,99,114,115,116,117,118,120,123,124,125,126,127]. However, findings from animal studies and cell lines have not yet been validated in humans, and their clinical significance remains unknown.

The third major area concerns metabolic diseases, where numerous studies indicate a potential link between 1,25(OH)_2_D and the regulation of pathways involved in glucose and lipid metabolism, metabolic dysfunction-associated steatotic liver disease (MASLD), and insulin resistance [107,108,109,110,111]. In some interventional studies, improvements in metabolic parameters have been observed, which were associated with changes in the expression of selected miRNAs [112,113].

Another important area of research concerns the prenatal period and developmental programming. Evidence from experimental studies, primarily in animal models, suggests that maternal vitamin D deficiency may lead to persistent epigenetic alterations in offspring, including changes in DNA methylation, increased adipogenesis, impaired glucose metabolism, enhanced inflammatory responses, hypertension, and alterations in skeletal development [70,71,72,74]. These findings indicate a potential role for vitamin D in long-term phenotypic programming; however, they require validation in human studies.

With regard to the skeletal system, available experimental evidence suggests that 1,25(OH)_2_D may be involved in the epigenetic regulation of genes associated with bone mineralization and development, including *Rxra*, *Runx2*, and *Osterix* [73,80]. However, findings from human studies remain less consistent and conclusive compared to those obtained from experimental models [80,81,82].

Similarly, in the context of epigenetic aging, some studies indicate a potential association between higher 25(OH)D concentrations or vitamin D supplementation and a lower epigenetic age. However, these findings are inconsistent and do not allow for definitive conclusions [62,63,64,65,66,67,68,83,84].

A key aspect of the present analysis is the comparison of the strength of evidence across different study types. Experimental studies provide the most compelling mechanistic insights into the action of 1,25(OH)_2_D and support its role as an epigenetic modulator. Observational and population-based studies further support these findings, demonstrating associations between 25(OH)D concentrations and DNA methylation patterns as well as other epigenetic markers; however, due to their design, they do not allow for the establishment of causal relationships.

Interventional studies, including randomized controlled trials (RCTs), are of the greatest clinical relevance, although their results remain heterogeneous. In some studies, changes in miRNA expression, methylation of selected loci, and epigenetic age markers have been reported [62,63,67,68,78,80,83,102,112,113,129,133,140], whereas global changes in DNA methylation have been less frequently observed [60]. These findings suggest that the effects of 1,25(OH)_2_D are more tissue-specific and locus-specific rather than systemic and uniform.

In summary, the available evidence suggests that 1,25(OH)_2_D may play a significant role as an epigenetic modulator, influencing gene expression at multiple levels. Its best-documented effects relate to the immune system, inflammatory processes, carcinogenesis, metabolism, and prenatal programming. The strongest evidence derives from experimental studies, whereas clinical data, although promising, remain less conclusive. The current state of knowledge supports considering 1,25(OH)_2_D as an important component of epigenetic regulation, while highlighting the need for further well-designed clinical trials to clarify the scope and clinical relevance of these mechanisms.

## 7. Individual Response to Vitamin D Supplementation

One of the major challenges in evaluating the biological and clinical effects of vitamin D supplementation in vivo is the substantial interindividual variability in serum 25(OH)D responses. Although a dose–response relationship exists between cholecalciferol intake and circulating 25(OH)D concentrations, this relationship is approximately linear at daily doses of 1000–2000 IU and gradually plateaus at higher doses. Importantly, the dose required to achieve sufficient serum 25(OH)D concentrations depends strongly on baseline 25(OH)D status [149].

Serum 25(OH)D levels are influenced by both genetic and environmental factors, including polymorphisms in genes involved in vitamin D metabolism, ultraviolet B (UVB) exposure, dietary intake, age, infections, and environmental pollutants such as cigarette smoke [33]. Genome-wide association studies (GWAS) have identified single-nucleotide variants in genes involved in vitamin D metabolism, including *DHCR7*, *CYP2R1*, and *GC*, which collectively explain only a small proportion of the variance in circulating 25(OH)D levels. Polymorphisms in *DHCR7*, *VDR*, and VDR target genes such as *CD14* and *CARD9*, prevalent in European populations, may contribute to greater sensitivity to vitamin D compared with African and Asian populations [150]. A large GWAS involving nearly 80,000 participants demonstrated that common single-nucleotide polymorphisms account for approximately 7.5% of the variance in serum 25(OH)D concentrations [151].

Genetic variants (SNPs) within genes involved in the vitamin D pathway have been described as potential modulators of epigenetic regulation. However, their effects appear to be primarily indirect and are thought to result from modulation of *VDR* gene expression, promoter activity, transcript stability, receptor transcriptional activity, and the availability of active vitamin D metabolites [152].

A variant located in the promoter region, *Cdx2* (rs11568820), may reduce the promoter activity of the VDR gene [153]. In contrast, the *VDR* variant known as *FokI* (rs2228570) influences the production of different receptor isoforms and alters their transcriptional activity [33,154].

The *BsmI* (rs1544410), *ApaI* (rs7975232), and *TaqI* (rs731236) variants may also participate in the regulation of VDR gene expression [152]. Among these, the strongest direct evidence linking SNPs with epigenetic mechanisms concerns *TaqI* (rs731236) and *VDR* gene methylation [155,156]. The *TaqI* and *FokI* variants might be associated with a better response to vitamin D supplementation [157].

It also appears that genetic variants in the *GC* gene (including rs2282679, rs4588, and rs7041), as well as in *CYP27B1* and *CYP24A1*, may indirectly modulate VDR-dependent epigenetic responses through effects on the bioavailability, activation, or degradation of vitamin D metabolites [152]. However, it should be emphasized that most data regarding the relationships between vitamin D, SNPs, and epigenetics are associative in nature and therefore do not allow for definitive conclusions regarding causal relationships.

As discussed above, individual differences in serum 25(OH)D levels and responses to cholecalciferol supplementation may also be influenced by epigenetic variation, particularly DNA methylation of genes encoding enzymes involved in vitamin D metabolism. Non-responders to cholecalciferol supplementation exhibit higher baseline methylation of *CYP2R1* and *CYP24A1*, but not after intervention, which may explain the attenuated increase in serum 25(OH)D observed in this group. Increased methylation of *CYP2R1* may impair cholecalciferol activation, while similar levels of *CYP24A1* methylation may maintain vitamin D catabolism, resulting in reduced net responsiveness [102]. In a predictive model incorporating DNA methylation, vitamin D intake, calcium intake, and cumulative UV exposure, inclusion of methylation levels at *CYP2R1*, *CYP27B1*, *CYP24A1*, and *VDR* significantly improved the prediction of plasma 25(OH)D concentrations. Notably, *CYP2R1* methylation emerged as an independent negative predictor, whereas *VDR* methylation was a positive predictor of circulating 25(OH)D [98].

Carlberg and colleagues proposed the concept of a personal vitamin D response index, which reflects the efficiency of the molecular response to cholecalciferol supplementation and may differ substantially from the static assessment of vitamin D status based solely on serum 25(OH)D levels [158]. This concept is based on a series of vitamin D intervention trials, including VitDbol, VitDHiD, and VitDmet, in which healthy individuals received either a single high-dose bolus of cholecalciferol (80,000 IU) or repeated lower doses over extended periods of up to 5 months [41]. These studies primarily focused on early molecular responses in PBMCs collected before and after supplementation [159]. PBMC molecular changes may serve as accessible pharmacodynamic biomarkers of 1,25(OH)_2_D activity. However, the restriction to a single cell type raises questions regarding the extent to which these findings can be extrapolated to other cell types and tissues in which 1,25(OH)_2_D exerts its regulatory functions. Moreover, the reported molecular responses do not necessarily translate into clinically meaningful outcomes. The VitDbol and VitDmet trials enabled stratification of participants into high, mid, and low responders based on their molecular responses. In the five-month VitDmet study, 24 VDR target genes were identified as potential markers of vitamin D responsiveness, alongside 12 biochemical and clinical parameters. Given the prolonged duration of the intervention, these responses were suggested to be largely mediated by epigenomic mechanisms. Importantly, several VDR target genes identified in VitDmet were also confirmed as markers of rapid transcriptional response in the acute VitDbol study [160]. Both trials consistently classified approximately 25% of participants as low responders. High responders may tolerate lower 25(OH)D serum levels and require less supplementation, whereas low responders may benefit from higher doses, as their baseline 25(OH)D concentrations may be insufficient to meet individual physiological demands [150].

The VitDbol study protocol was replicated in a Saudi cohort (*n* = 100). Among the participants, 39% were classified as low responders, which may reflect reduced evolutionary pressure against low vitamin D responsiveness in regions with sufficient sunlight exposure for endogenous cholecalciferol synthesis. Obesity was identified as a potential confounding factor limiting the utility of pro-inflammatory cytokines as biochemical markers for establishing a personalized vitamin D response index. A similar limitation may also apply to other conditions associated with chronic inflammation. Similarly to the VitDbol and VitDmet studies, the interpretation of these findings is limited by the relatively small sample size and lack of randomization [161]. A randomized controlled trial supported the concept of an individual genomic response to cholecalciferol supplementation that appears to be independent of the increase in serum 25(OH)D levels. However, the study was limited by its small sample size and the assessment of genomic responses exclusively in peripheral white blood cells. Notably, approximately 30% of participants were identified as low responders, which is consistent with the findings of the VitDmet and VitDbol studies [162].

It has therefore been proposed that low responders may require higher daily doses of cholecalciferol to achieve optimal hormonal activity and maximal disease-protective effects. Consequently, personalized cholecalciferol supplementation strategies, guided by an individual vitamin D response index and personalized optimal 25(OH)D serum level, may be more appropriate than uniform population-based recommendations [158]. However, the current evidence base remains limited by small sample sizes, lack of randomization, and identified limitations such as comorbidities. Moreover, it is still unclear whether interindividual differences in the vitamin D response index translate into clinically meaningful outcomes. Combined with the cost and technical complexity of establishing a personalized vitamin D response index in clinical practice, these limitations currently preclude its routine clinical implementation.

## 8. Limitations

The narrative nature of this review should be acknowledged, as it may contribute to potential selection bias. We have extensively referenced the work of Carlberg and colleagues across the manuscript. We would like to acknowledge their valuable contribution to this field.

The main limitation is that the majority of studies are cell culture and animal models, with a modest number of observational studies and even fewer randomized controlled trials. Therefore, the clinical significance remains limited, and the findings mainly indicate a mechanistic basis and suggest directions for future research.

Another significant limitation of the analyzed studies is the heterogeneity of study populations, including differences in ethnicity, genetic background, and environmental exposures. These factors may influence vitamin D metabolism, baseline 25(OH)D concentrations, and epigenetic responses, thereby limiting the comparability of the results. Furthermore, differences in age between study groups, as well as the presence of comorbidities, may affect epigenetic assessments, particularly in the context of epigenetic aging markers and the dynamics of DNA methylation changes.

Additional complexity arises from interindividual differences in cutaneous cholecalciferol synthesis, dietary intake, and evidence suggesting that complete biological equivalence between oral supplementation and dermal synthesis cannot be assumed [6]. Moreover, individual variability in molecular response to cholecalciferol supplementation further complicates the interpretation of supplementation studies based solely on serum 25(OH)D levels. The concept of a personal vitamin D response index suggests that, in some individuals, commonly used supplementation doses may be insufficient to elicit measurable molecular and, presumably, clinical effects [150]. However, further evaluation is needed before these findings can inform clinical decision-making and recommendations.

Another important limitation is the absence of baseline serum 25(OH)D measurements in some studies, despite evidence that the effects of cholecalciferol supplementation are more pronounced in individuals with vitamin D deficiency. Indeed, RCTs conducted in vitamin D-replete populations have generally failed to demonstrate additional health benefits or improvements in clinical outcomes following supplementation [5,150].

Vitamin D supplementation is included in public health recommendations in many countries, particularly during periods of limited sunlight exposure [158]. However, establishing clear and universally applicable dosing guidelines remains challenging, as the optimal target serum 25(OH)D concentration for disease prevention is still debated [158,163,164]. The range most commonly considered adequate is 75–100 nM (30–40 ng/mL) of 25(OH)D [150]. In experimental studies, vitamin D and its metabolites are often administered at doses or routes that differ substantially from typical human exposure and under conditions that do not fully reflect the complexity of vitamin D metabolism and regulation in vivo [159]. In contrast, doses and supplementation regimens used in RCTs vary substantially. Another important consideration is the heterogeneity of vitamin D exposure analyzed in observational studies, including serum 25(OH)D levels, dietary vitamin D intake, and supplementation of various forms of vitamin D, which, combined with the inability to establish causality, limits the clinical implications of these findings.A further limitation of human studies is the restricted range of cell types available for genomic and epigenomic analyses, primarily immune cells. Most experimental studies included peripheral blood lymphocytes, macrophage-like cells, hepatic stellate cells, colorectal cancer cells, and the monocytic THP-1 cell line [10,76]. While these models are experimentally accessible, they do not allow comprehensive characterization of cell-specific VDR cistromes or epigenetic regulation in primary 1,25(OH)_2_D target tissues.

A limiting factor in the analysis and interpretation of epigenetic studies related to vitamin D may be methodological heterogeneity associated with the techniques used to assess DNA methylation, which can affect the sensitivity, scope of analysis, and reproducibility of results. In addition, DNA methylation patterns exhibit high tissue specificity and may vary depending on age, disease status, environmental exposures, and the cellular composition of samples. Similar limitations apply to methods used for assessing chromatin accessibility. Consequently, methodological differences across studies may reduce the comparability of findings and complicate the interpretation of causal relationships between vitamin D signaling and epigenetic regulation.

Finally, not all studies incorporate parallel analyses of transcriptomic and proteomic outcomes, which limits the biological interpretation of observed epigenetic changes. Importantly, not all epigenomic alterations translate into changes in gene expression or protein production [41,160].

Another area of vitamin D research that requires deeper investigation concerns its rapid non-genomic actions, which may influence downstream genomic and epigenetic regulation. These responses involve membrane-associated VDR signaling; however, accumulating evidence suggests functional cooperation between membrane and nuclear VDR pathways. Through the activation of second messengers and kinase cascades, including MAPK, PKC, and PI3K signaling pathways, 1,25(OH)_2_D can modulate transcriptional programs as well as chromatin-related regulatory mechanisms [165,166].

Another promising area for further investigation is the role of RNA modifications in vitamin D signaling. Among RNA modifications, that of N6-methyladenosine (m6A) is the most abundant and plays a key role in RNA stability and translation. Increasing evidence suggests that m6A modification may participate in the post-transcriptional regulation of VDR expression. Previous studies have demonstrated that VDR mRNA stability may also be regulated by miRNAs, indicating the existence of multiple post-transcriptional regulatory checkpoints capable of modulating VDR levels and, consequently, cellular responsiveness to vitamin D. However, the direct mechanisms linking vitamin D signaling with RNA methylation pathways remain insufficiently understood and require further investigation [167,168].

Overall, there remains a need for adequately powered, long-term, well-designed randomized controlled trials that evaluate cholecalciferol supplementation as the primary intervention and include predefined hard clinical endpoints [169].

## 9. Conclusions

Experimental studies using cell cultures and animal models provide mechanistic evidence that 1,25(OH)_2_D exerts pleiotropic biological effects mediated by epigenetic regulation of pathways involved in immune regulation, metabolism, and cancerogenesis. However, the translation of these molecular effects into clinical benefit remains limited and inconsistent, partly due to the limited number of randomized controlled trials and observational studies specifically evaluating cholecalciferol supplementation rather than serum 25(OH)D levels.

The main challenge in designing cholecalciferol supplementation studies lies in the complex relationship between the intervention itself, the resulting biological effects, and the clinical endpoints. In particular, vitamin D supplementation studies are complicated by the interplay between oral cholecalciferol supplementation, the commonly used marker of vitamin D status (serum 25(OH)D concentration), and the biologically active metabolite 1,25(OH)_2_D, including its molecular effects and clinical relevance.

Unlike most drugs, cholecalciferol administered in interventional studies is not the sole source of exposure for participants, as additional vitamin D is derived from dietary intake and cutaneous synthesis. This limitation could be partially reduced by conducting studies during winter months or in geographical locations with limited sun exposure, as well as by including detailed dietary questionnaires in the study design. Baseline serum 25(OH)D concentration should also be established, as supplementation appears to be most beneficial in individuals with vitamin D deficiency. A potentially useful strategy may involve adjusting cholecalciferol doses to achieve a target serum 25(OH)D concentration [170]. However, this does not directly translate into changes in 1,25(OH)_2_D levels or biological effects. Another proposed improvement is the inclusion of longer follow-up periods, as clinical effects may develop over time [171].

Available interventional studies suggest that future research should focus on specific molecular endpoints rather than global methylation changes or overall miRNA profiles. Mechanistic findings from cell culture and animal studies may help identify such candidate molecular endpoints. One promising area is epigenetic aging; however, standardization of the epigenetic clocks used would be essential, preferably through the application of second- and third-generation epigenetic clocks [172].

Another important issue is the substantial interindividual variability in responsiveness to cholecalciferol supplementation, including differences in molecular responses to 1,25(OH)_2_D. The concept of a personal vitamin D response index offers promise for personalized supplementation strategies and optimization of clinical outcomes; however, it is not currently a validated clinical assay. The bolus supplementation strategy, used in studies such as the VitDbol trial, is easier to implement but may not accurately reflect the biological effects of daily supplementation. Therefore, long-term daily supplementation protocols may be more appropriate [173]. Assessment of the personal vitamin D response index should not be considered a screening tool but may potentially be useful in selected groups of patients, for example, those with absent or inadequate clinical responses to cholecalciferol supplementation despite normal serum 25(OH)D levels. In addition, a personal vitamin D response index based solely on biochemical parameters, such as pro-inflammatory cytokine profiles, may be inadequate in patients with chronic inflammation, including individuals with obesity. In such cases, assessment of the expression of VDR target genes could be considered [174], although this approach may be neither cost-effective nor practical in routine clinical settings. A potentially more feasible link between static serum 25(OH)D measurements and advanced PCR-based gene expression analyses may be the assessment of serum PTH concentrations, although this approach also has important limitations.

Overall, current evidence from cell culture and animal studies supports 1,25(OH)_2_D as an important epigenetic modulator. Nevertheless, these observations require confirmation in well-designed studies integrating molecular endpoints with clinically meaningful outcomes.

## Figures and Tables

**Table 2:** Summary of key findings on epigenomic effects of vitamin D and its active metabolites’ stimulation from cell culture studies.

No.	Study Subject	Intervention/Exposure	Epigenetic Effect	Phenotypic Effect	Overall Conclusions
1	SW480-ADH colon cancer cell culture	1,25(OH)_2_D stimulation	↑ *LSD1*, *LSD2*, *JARID2*, *KDM5B*; ↓ *KDM4A/C/D*, *KDM5A*, *KDM2B*, JMJD5/6 expression	Not tested (in other studies, breast cancer cell proliferation, angiogenesis)	1,25(OH)_2_D selectively reshapes the demethylase landscape [20]
2	MCF-10A cell culture (non-tumorigenic epithelial cell line); RWPE-1 adherent human prostate epithelial cells from healthy male (*n* = 1)	1,25(OH)_2_D stimulation; *HDAC4/6* silencing	Cyclical VDR recruitment to VDRE; ↑ *HDAC4/6* expression; histone H4 deacetylation in *IGFBP3* gene	IGFBP3 transcription	HDAC4/6 contribute to VDR-mediated chromatin regulation [44]
3	Human acute monocytic leukemia cell line (THP-1)	1,25(OH)_2_D stimulation; HDAC inhibitor exposure	Increase in chromatin accessibility, especially in DR3	VDR peaks in genes involved in cell growth and immunity	1,25(OH)_2_D opens chromatin in VDR binding loci, which may include H3K27 acetylation [46]
4	Human acute monocytic leukemia cell line (THP-1)	1,25(OH)_2_D stimulation	Changes in H3K4me3 (promoters) and H3K27ac (enhancers) across numerous genomic regions	Identification of 59 vitamin D target genes	1,25(OH)_2_D modifies the human epigenome via histone modification [51]
5	Murine mesenchymal stem cells	1,25(OH)_2_D stimulation	↑ H3K9 acetylation at 1,25(OH)_2_D-regulated genes	mRNA transcript regulation	Epigenetic regulation may occur outside TSS regions [52]
6	Human dendritic cells	1,25(OH)_2_D stimulation	VDR interaction with TET2 → demethylation of VDR binding sites	Altered immune gene expression	1,25(OH)_2_D induces locus-specific DNA demethylation [55]
7	Peripheral blood mononuclear cells (PBMCs) from healthy adults *n* = 4)	1,25(OH)_2_D stimulation	No substantial genome-wide methylation changes	None	Effects might be cell type- and loci-specific [58]
8	Colon cancer cell lines (SW480-ADH and HCT116)	1,25(OH)_2_D stimulation	↑ miR-22 (in a time-, dose-, and VDR-dependent manner)	↓ cell proliferation, migration; suppression of *NELL2*, *OGN*, *HNRPH1*, *RERE* and *NFAT5* genes	1,25(OH)_2_D exerts its anti-tumor effect by induction of miR-22. Lower expression of miR-22 in CRC patients [114].
9	Prostate cancer cell line (LNCaP)	1,25(OH)_2_D stimulation	↑miR-98 expression; ↑ miR-98 blood level in mice	Anti-growth effect	miR-98 provides potential therapeutic target and potential biomarker [117]
10	Prostate cancer cell line (LNCaP)	1,25(OH)_2_D + testosterone stimulation	↑ miR-22, miR-29ab, miR-134, miR-1207-5p, miR-371-5p ↓ miR-17, miR-20a	mRNA products of genes implicated in cell cycle progression, lipid metabolism, and calcium homeostasis are targets of these micro-RNAs.	Decline in testosterone and 1,25(OH)_2_D with age is suggested to be implicated in prostate cancer oncogenesis [118].
11	Breast epithelial cell line (MCF12F)	25(OH)D stimulation (250 nmol/L) under stress conditions	Reversed stress-induced miR-182 alteration	Cell protection against cell death (↓ p53 expression and ↓ PCNA).	Protective role of 25(OH)D against stress might be mediated by modified miRNA expression [120]
12	Breast cancer cell lines	1,25(OH)_2_D treatment	↓ methylation of *CDH1* promoter	↑ e-cadherin expression	A novel mechanism for the action of 1,25(OH)_2_D in breast cancer cells [93].
13	Cancer cell lines (OV2008, CAOV3, MCF-7, Ishikawa, HeLa cells) and human borderline and malignant ovarian tumor tissues	1,25(OH)_2_D stimulation	↑ miR-498 expression	↓ mRNA expression of the human telomerase reverse transcriptase—suppression of ovarian cancer growth	Based on effect of 1,25(OH)_2_D stimulation, decreased miR-498 expression in tumor samples and compromised effect after MiR-489 depletion; MiR-489 is identified as important mediator of 1,25(OH)_2_D anti-tumor action [123].
14	Leukemia cell lines (MV-4-1, Thp-1, HL-60, K562, KG-1) and lymphoma cell lines (Raji, Daudi, Jurkat, U2932)	1,25(OH)_2_D, tacalcitiol stimulation	↑ miR-27b and miR-125b, diverse effect on miR-181a and miR-181b	Regulation of p27, Bak1, NFκB, and CYP24A1 proteins	MiRNAs might mediate anticancer activity of active forms of cholecalciferol in leukemias and lymphomas [124].
15	Head and neck cancer (HNC) cell lines (CAL-27, SCC-25)	Cholecalciferol stimulation	↓ hsa-miR-7-1, hsa-miR-335 ↑ hsa-miR-331-5p, hsa-miR-616, hsa-miR-632	Modulation of multiple pathways relevant to HNC including MAPK, PI3K, RAS, chemokine signaling, negative regulation of cellular proliferation, angiogenesis; canonical WNT signaling and cell adhesion.	Cholecalciferol induces changes in miRNAs patterns and protein function in HNC [126].
16	Cancer cell lines (Kasumi-1, MDA-MB-231)	1,25(OH)_2_D stimulation	↑ miR-302c, miR-520c expression	Increased susceptibility to NK92 cells.	1,25(OH)_2_D anti-tumor role can be mediated by miRNAs and stimulation of immune system [128].
17	Endothelial progenitor cells (EPCs) treated with high levels of glucose	1,25(OH)_2_D stimulation	Altered expression of hundreds of mRNAs, lncRNAs circRNAs, and miRNAs	Associated with MMP and GTPase activities, specific signaling pathways, and components of actin, extracellular matrix, or adherens junction	Improved cellular function via ceRNA networks [104]
18	Human model of SGBS (Simpson–Golabi–Behmel syndrome) adipocytes—extracellular vesicles (EVs)	1,25(OH)_2_D stimulation	Prevented miR-146a and miR-155 upregulation in cells, and miR-155 in EV	↓ inflammation	Suggests new mechanism of 1,25(OH)_2_D anti-inflammatory action [106].
19	DENV-2 infected monocyte-derived macrophages	1,25(OH)_2_D stimulation	↓ miR-182-5p, miR-130a-3p, miR-125b-5p, miR-146a-5p, and miR-155-5p	↑ IFN-I, OAS1, and SOCS1; ↓ reduced production of pro-inflammatory cytokines (TNF-α, IL-6) and decreased expression of TLR9	1,25(OH)_2_D modulates the inflammatory response during DENV-2 infection by inhibiting specific miRNAs, which promotes an antiviral state and prevents exacerbated inflammation without directly inhibiting viral replication [130].
20	Human bronchial fibroblasts	1,25(OH)_2_D stimulation	↓ miR-21-5p	Inhibition of TGF-β/Smad signaling and fibrotic remodeling	1,25(OH)_2_D mitigates airway fibrosis via miRNA-mediated pathway inhibition [135].
21	Human lung fibroblasts	1,25(OH)_2_D stimulation	↓ miR-27b	Impact on fibrotic processes	1,25(OH)_2_D targets miRNAs involved in lung tissue remodeling [136].
22	Human placental tissue	1,25(OH)_2_D stimulation	↑ miR-181b-5p and miR-26b-5p	Inhibition of CRH and COX-2	1,25(OH)_2_D-regulated miRNAs may protect against preterm labor [138]
23	Human osteoblasts	1,25(OH)_2_D stimulation	Modulation of miR-1228 and miR-637	Targeting of *BMP2K* and *COL4A1*	1,25(OH)_2_D regulates bone-related gene targets through specific miRNAs [139]
24	Monocyte-derived macrophages	1,25(OH)_2_D stimulation	↓ miR-6501-3p, miR-1273h-5p, miR-665, miR-1972, miR-1183, and miR-619-5p	Regulation of *PDCD1LG2*, *IL1B*, and *CD274* expression	1,25(OH)_2_D modulates innate immunity via specific miRNA downregulation [129]
25	Human acute monocytic leukemia cell line (THP-1)	1,25(OH)_2_D ± LPS stimulation	↑ chromatin accessibility	Enhanced transcriptional response	Immune activation amplifies 1,25(OH)_2_D effect [142].
26	Human acute monocytic leukemia cell line (THP-1)	1,25(OH)_2_D stimulation	Modulation of ~2000 CTCF sites; TAD remodeling	Broad gene regulation	3D chromatin architecture central to 1,25(OH)_2_D action [144].
27	Human breast cancer cells (MDA-MB453)	1,25(OH)_2_D stimulation	Cyclical chromatin looping between distal VDREs and the *p21* TSS; ↑ H3K9 acetylation; LSD1-dependent histone demethylation; dissociation of NCoR1/HDAC co-repressor complexes	↑ *p21*/CDKN1A transcription	1,25(OH)_2_D regulates p21 transcription through cyclical chromatin looping dependent on histone acetylation and demethylation [145]
28	RWPE1 human non-malignant prostate epithelial cells; RWPE2, PC3, DU145 prostate cancer cell lines	1,25(OH)_2_D stimulation	↑ VDR binding to functional VDREs within the *KLK* locus; ↑ CTCF occupancy at insulator regions; chromatin decondensation associated with H4 acetylation near the *KLK6* promoter	↑ *KLK5/6/7/8/9* expression in non-malignant prostate cells; reduced or absent induction in prostate cancer cell lines	1,25(OH)_2_D regulates the *KLK* gene cluster through VDR-dependent chromatin organization and CTCF insulator occupancy in prostate cells [146].

↑ increase/upregulation ↓ decrease/downregulation.

**Table 3:** Summary of key findings on epigenomic effects of cholecalciferol supplementation from animal model studies.

No.	Study Subject	Intervention/Exposure	Epigenetic Effect	Phenotypic Effect	Overall Conclusions
1	Rat experimental immune encephalomyelitis model (*n* = 29); CD4^+^ T lymphocytes	Cholecalciferol supplementation 10 IU vs. 0 IU	↓ HDAC1, HDAC2, KDM1B, KDM2A, KDM5A, ↓ DNMT1, DNMT3a, DNMT3b → global reduction in DNA methylation ↑ snRNA, miRNA, ribosomal RNA expression	↓ Th1/Th17 proliferation and activity	Cholecalciferol exerts immunomodulatory effects via epigenetic pathways including histone modifications, DNA methylation, ncRNA expression [45]
2	Rats with DM2 (*n* = 32); liver tissue	High-dose cholecalciferol vs. moderate dose vs. aerobic training vs. control	↓ miR-122, miR-9	Beneficial effect on insulin levels, insulin resistance, and lipid metabolism	Both high-dose cholecalciferol supplementation and aerobic training improved metabolic outcomes through modulated miRNA expression [107].
3	Rats with DM2 (*n* = 40) vs. control (*n* = 10); heart tissue	Aerobic training (AT) + Vitamin D injection, AT, Vitamin D injection, control	↑ VEGF-B, IGF-1, ↓ miR-1 in AT + vitamin D group	Decreased body weight, BMI, food intake in all intervention groups	Cardio-protective effect of AT and vitamin D mediated partially by miRNA [108]
4	Rats after myocardial infarction (*n*= 48)	Aerobic-resistance training (ART), cholecalciferol supplementation, combination of both	↓ miRNA-15a, miRNA-146a ↑ VEGF, PI3K, and eNOS expression	Highest ejection fraction, fractional shortening, exercise capacity and maximal load test, increased angiogenesis were observed in the groups treated with cholecalciferol, ART, and cholecalciferol + ART.	Combination of ART and cholecalciferol supplementation proved to be most effective in improvement of cardiac function of rats after MI [109].
5	Rats (*n* = 45)	HFD (high-fat diet), HFD + vitamin D supplementation in drinking water (20,000 IU/kg/week), LFD (low-fat diet), LFD + vitamin D, control (standard diet)	Modulation of miR-155/TLR7 axis	Improved lipid profile, transaminases, inflammatory factors	Vitamin D supplementation attenuated metabolic effects of HFD [110].
6	Rats (*n* = 24)	Fatty liver + cholecalciferol supplementation group, experimental fatty liver group, control	↑ increased miR-33a and miR-200c compared to the fatty liver group	Improved transaminases, TNF-α, and malondialdehyde levels and reduced liver steatosis compared to the fatty liver group, but not to control levels.	Cholecalciferol supplementation attenuated fatty liver disease metabolic and histopathological disturbances [111].
7	Swine (*n* = 48)	Oral supplementation for 88 days 1. standard dose of cholecalciferol (2000 IU/kg) 2. standard dose of calcidiol 3. increased dose (3000 IU/kg) of cholecalciferol 4. cholecalciferol + calcidiol combination	Cholecalciferol + calcidiol supplementation altered multiple lung miRNAs (miR-150, miR-193, miR-145, miR-574, miR-340, miR-381, miR-148, miR-96); increased cholecalciferol dose affected only miR-215, while calcidiol showed no significant miRNA effects despite the greatest increase in 25(OH)D	Regulation of pathways linked to inflammation and asthma	Possible interplay between different vitamin D metabolites in modulating miRNA expression [137].

↑ increase/upregulation ↓ decrease/downregulation.

**Table 4:** Summary of key findings on the epigenetic effects of vitamin D and its metabolites, including stimulation, supplementation, and serum levels, across diverse studies.

Study Type	Study Subject	Intervention/Exposure	Epigenetic Effect	Phenotypic Effect	Overall Conclusions
Experimental (cell culture + animal model)	Porcine intestinal epithelial cell line (IPEC-J2)	1,25(OH)_2_D stimulation followed by RV infection	Repression of rotavirus-induced miR-155-5p up-regulation	Activation of the TBK1/IRF3 signaling pathway and increased IFN-β production.	1,25(OH)_2_D alleviates rotavirus infection by suppressing the expression of miR-155-5p, which directly targets and inhibits TBK1, thereby restoring the host’s innate antiviral IFN-I response [131].
		Cholecalciferol oral supplementation (5000 IU/kg vs. 200 IU/kg) for 21 days, followed by RV challenge		Attenuation of RV-induced diarrhea and preservation of intestinal morphology	
Experimental (cell culture + animal model)	Human adipocytes; 3T3-L1 mouse adipocytes	1,25(OH)_2_D stimulation	↓ miR-146a, miR-150, miR-155	↓ inflammation	1,25(OH)_2_D attenuates inflammation in adipose tissue through regulation of miRNAs [105]
		Diet supplemented with cholecalciferol (3000 IU/kg of body weight) vs. not supplemented (300 IU/kg of body weight)			
Experimental (cell culture + animal model)	Colon cancer cell line (HT-29)	1,25(OH)_2_D stimulation	↑ miR-627—JMJD1A downregulation—↑ H3K9 methylation—↓ expression of proliferative factors (GDF15)	↓ cell proliferation, xenograft growth in mice	An example of interaction of miRNA and histone modifications stimulated by 1,25(OH)_2_D [115]
		Intraperitoneal injection of 1,25(OH)_2_D			
Experimental (cell culture + animal model)	VDR-deleted keratinocytes	VDR depletion	↓ tumor suppressor lncRNAs (Kcnq1ot1, lincRNA-p21, Foxn2-as, Gtl2-as, H19-as) ↑ oncogenic lncRNAs (HOTTIP, Nespas, Malat1, mHOTAIR)	Not assessed.	1,25(OH)_2_D signaling, with VDR at its center, plays a protective role in skin cancer through the regulation of lncRNAs [127]
Experimental (cell culture + animal model + ex vivo human tissue)	Human iPSC-derived β cells; INS-1 cells	Calcipotriol treatment alone or combined with BRD9 inhibition	VDR-dependent switching of BRD9-containing ncBAF complexes to BRD7/PBAF complexes; altered β-cell identity and inflammatory transcriptional programs	↑ insulin secretion; ↑ β-cell survival; ↓ inflammatory and ER stress responses; improved glucose metabolism in diabetic mice	VDR activation protects β cells through switching of SWI/SNF-BAF chromatin remodeling complexes and restoration of β-cell transcriptional programs [147]
Mixed design (cell culture + observational)	Patients with chronic rhinosinusitis (CRS); Human nasal epithelial cells (HNECs)	25(OH)D, LPS and SEB stimulation in HNECs	↓ BRG1, BAF155, and INI1 after LPS stimulation; ↑ BAF170 and INI1 after SEB stimulation; ↑ BAF155 and BAF170 after 25(OH)D + SEB stimulation	↓ SWI/SNF subunit expression in CRS tissues; inverse correlation of BRG1 with eosinophils and neutrophils in rhinosinusitis with nasal polyps	SWI/SNF complex alterations may contribute to altered VDR- and steroid-related responses in chronic rhinosinusitis [148]
Mixed design (cell culture + animal model + observational)	Colon cancer cell lines (HT-29, DLD-1)	1,25(OH)_2_D stimulation + H19 overexpression	1,25(OH)_2_D ↓ expression of H19 trough VDR mediated C-Myc/Mad-1 network regulation H19 ↓ VDR through miR-675-5p	↓ cell proliferation	LncRNA H19 overexpression in CRC might be one of the mechanisms underlying the decreased expression of *VDR*, causing resistance to 1,25(OH)_2_D in vivo and in vitro [116].
		Intraperitoneal injection of 1,25(OH)_2_D + H19 overexpression			
Mixed-design study (cell culture + observational)	Colon cancer cell cultures	1,25(OH)_2_D stimulation; JMJD3 depletion in cell culture models	↑ KDM6B/JMJD3 expression (possibly via ↓ miR-200b/c [20])	Activation of tumor suppressor genes (e.g., *CDH1*); loss of protective effects after JMJD3 depletion	JMJD3 imight be a potential mediator of 1,25(OH)_2_D protective effects in colon cancer [43]
Mixed-design study (cell culture + cross-sectional)	Human colorectal carcinoma cell lines (HCT116)	Cholecalciferol stimulation	↓ *SFRP2* promoter methylation in tumor cells, but not in PBMCs or visceral adipose tissue in vitamin D-depleted group. No effect on *SFRP2* methylation (*SFRP2* promoter fully methylated) in HCT116	Not assessed.	Cell culture model results contradictory to CRC tissue obtained from patients. Higher 25(OH)D levels were associated with increased expression of *SFRP2*, a tumor suppressor gene [91].
		25(OH)D serum level			
Mixed-design study (experimental + RCT post hoc analysis)	Human placentas from healthy pregnancies (*n* = 6)	25(OH)D stimulation	Altered *RXRA* gene methylation	Increased *RXRA* gene expression in vitro	Despite *RXRA* gene methylation changes and increased expression in vitro, evidence from human study is not consistent [80]
		Maternal cholecalciferol supplementation 1000 IU/day vs. placebo		Modest association between *RXRA* CpG5 and CpG11 methylation and neonatal bone indices in placebo group but not in supplemented group	

↑ increase/upregulation ↓ decrease/downregulation.

**Table 5:** Summary of observational evidence linking vitamin D exposure (dietary intake, supplementation, and status) and epigenetic modifications.

No.	Study Type	Study Subject	Intervention/Exposure	Epigenetic Effect	Phenotypic Effect	Overall Conclusions
1	Observational	Australians > 65 years old (*n* = 80)	25(OH)D serum level	↓ *CYP2R1*, *CYP24A1* methylation, ↑ *VDR* methylation; *CYP27B1* not affected	Not assessed.	Possible feedback regulation of vitamin D metabolism [98].
2	Observational (cross-sectional)	African American adolescents with vitamin D deficiency (*n* = 11) vs. control (*n* = 11); leukocytes	25(OH)D serum level	↑ *CYP2R1* methylation; ↓ *CYP24A1* methylation in vitamin D-deficient group	Not assessed.	Severe vitamin D deficiency is associated with methylation changes including genes involved in its metabolism [97].
3	Observational (cross-sectional)	Pulmonary tuberculosis patients (*n* = 122) vs. control (*n*= 118)	25(OH)_2_D, 1,25(OH)_2_D serum levels	Significant association with *CYP27A1* gene methylation	1,25(OH)_2_D associated with treatment outcomes	However, no significant interaction between *CYP27A1* methylation, 1,25(OH)D level, and treatment outcome was established [101].
4	Observational	Participants in German Asthma Family Study (*n* = 384); peripheral blood lymphocytes	25(OH)D, 1,25(OH)_2_D serum level	↓ 25(OH)D serum level—weak association with ↑ *CYP27B1* methylation, none with *CYP24A1*	Not assessed.	No significant association [100]
5	Observational	Patients undergoing CABG (*n* =10)	25(OH)D serum level	25(OH)D < 25 nmol/L ↑ miR-21 in aorta	The lower the difference between miR-21 in aorta and internal mammary artery, the lower the bone density.	Vitamin D deficiency might be linked to atherosclerosis and osteoporosis [141]
6	Observational (case-cohort)	Women 35–74 years old, with family history of breast cancer (*n* = 50,884, including 1070 cases and 1277 subcohort)	25(OH)D serum level	Association with methylation in *RXRA*, *NADSYN1/DHCR7*, *GC*, *EPHX1*, *SPN*, *SMARCD2*	Postulated changes in vitamin D-related genes, immune function, or regulation of VDR.	Suggested potential modulation of breast cancer risk via epigenetic mechanisms [94]
7	Observational (cross-sectional)	Colorectal cancer patients (*n* = 57) vs. control group (*n* = 50); blood and visceral adipose tissue	25(OH)D serum level	↓ *DNMT3A* expression, ↓ *VDR* methylation	↓ CRP, NFκB1	Low 25(OH)D levels and higher adipose tissue VDR expression may be linked to VAT–CRC interplay through methylation changes [89]
8	Observational (cross-sectional)	Colorectal cancer patients (*n* = 55) vs. control group (*n* = 35); visceral adipose tissue	25(OH)D serum level	25(OH)D correlated with ↑ LINE-1 methylation	Higher LINE-1 methylation was connected with lower CRC risk	25(OH)D levels may be associated with genomic stability, which in turn may be related to anti-oncogenic effects [88]
9	Observational (cross-sectional)	Colorectal cancer patients (*n* = 57) vs. healthy subjects (*n* = 55)	25(OH)D serum level	*DKK1* promoter demethylation	↑ *DKK1* expression; ↓ cell proliferation in vitro	Decreased methylation at specific loci may be linked to vitamin D-related anti-tumor mechanisms [92]
10	Observational (cross-sectional)	Colorectal cancer patients (*n* = 61) vs. healthy individuals (*n* = 68); visceral adipose tissue	25(OH)D serum level	Higher 25(OH)D level associated with *VDR*, *CYP27B1*, *IL-6*, *IL-10* methylation and expression	*IL-6* expression connected to poor survival rate; however, *IL-6* methylation was associated with increased risk of CRC	IL-6 role in CRC might be mediated by 1,25(OH)_2_D epigenetic actions [99]
11	Observational (ex vivo)	Colorectal cancer patients (*n* =20); tumor tissue	25(OH)D serum level	Low 25(OH)2 associated with global hypomethylation in tumor tissue	Not assessed	25(OH)D status has been linked to methylation changes in CRC [86]
12	Observational (ex vivo)	Healthy subjects (*n* =185); rectal biopsies	25(OH)2 serum level	↓ *APC* methylation; ↑ LINE-1 methylation	Not assessed	25(OH)D) may be associated with methylation changes in CRC, with a stronger association observed in females [87]
13	Observational	Colorectal cancer patients (*n*= 992)	Vitamin D intake (dietary sources, supplementation)	↓ *DKK1*, *WNT5A* methylation (however, not consistent among study locations and tumor stages)	Not assessed	A potential mechanistic link between vitamin D intake (primarily from dietary sources) and CRC protection [90].
14	Observational (cross-sectional)	HNSCC patients prior to treatment (*n* = 67)	Vitamin D dietary intake	↑ miR-375 expression	Not assessed	The results indicate potential associations involving oncogene c-MYC [125]
15	Observational	Mother/newborn pairs	Vitamin D supplementation	Lower epigenetic age acceleration	Epigenetic age acceleration was associated with aortic intima-media thickness in preterm infants; not associated with heart rate variability	Modest clinical effect, no causation established due to study type [84].
16	Observational (cross-sectional) study with an interventional component	Healthy Caucasians and African Americans aged 14–18 (*n* = 454); RCT—subgroup of vitamin D-deficient young African Americans, overweight/obese (*n* = 58); leukocytes	25(OH)D serum level; RCT Cholecalciferol monthly oral dose 18,000 IU/60,000 IU/120,000 IU/placebo for 16 weeks	Positive correlation between %5-mC and 25(OH)D level in African American group ↑ Global, dose-dependent 5–mC	Not assessed	Low 25(OH)D is linked to global hypomethylation in African Americans; Cholecalciferol supplementation dose-dependently increases global DNA methylation in deficient young individuals [62].
17	Observational cohort study with an association analysis	Longitudinal cohort from Berlin Aging Study II included in in the GendAge study (*n* = 1036)	Self-selection of vitamin D supplementation + successful treatment of vitamin D deficiency	Association between 25(OH)D levels and 7-CpG and GrimAge DNAmAA. ↓ 7-CpG and Horvath DNAmAA Horvath DNAmAA in vitamin D deficient participants who started supplementation compared to untreated vitamin D-deficient participants.	Not assessed	Participants with treated vitamin D deficiency do not differ in DNAmAA from vitamin D-sufficient participants [63]
18	Population-based	Adults from Aging Berlin Study II (*n* =1649)	25(OH)D serum level	Lower DNAmAA with sufficient 25(OH)D serum level	No association with Fried frailty score or functional assessment	Higher 25(OH)D serum level associated with reduced epigenetic age acceleration [64].
19	Population-based	Adults aged 50–74 years old (*n* = 9940 participants)	25(OH)D serum level	No association with telomere length or DNAmAA	Not assessed	25(OH)D suggested as a marker of aging independent of epigenetic indicators [65].
20	Population-based	Elderly Swedish cohort (*n* = 277); saliva samples	Total vitamin D intake (dietary sources, supplementation)	No global DNA methylation change; *SLC25A24* differential methylation	Not assessed	Limited global effect; locus-specific changes possible [61].
21	Population-based	South Australian healthy adults (*n* = 208); lymphocytes	UV exposure, 25(OH)D serum level	Inverse association between solar UV radiation and LINE-1 DNA methylation; 25(OH)D not independently associated	No clear phenotype	Secondary mechanisms can be responsible for 25(OH)D protective role in UV-induced damage [59].
22	Population-based	Mother/newborn pairs from PACE consortium (*n*= 3239) Cord blood DNA	Maternal 25(OH)D serum level during pregnancy	No association with global methylation, no consistent gene-specific methylation	Not applicable	Inconsistent findings [75].
23	Population-based	African American (*n* = 112) and European American mother/newborn pairs (*n* = 91); neonatal cord blood	Maternal 25(OH)D serum level during pregnancy	CpG methylation influenced jointly by ancestry and 25(OH)D serum level	Not assessed	Ancestry-specific DNA methylation patterns may be influenced by 25(OH)D serum level [76].
24	Population-based	Mother/child pairs over 3 years (*n* = 476)	Maternal 25(OH)D serum level during pregnancy	No association between maternal 25(OH)2 serum level and methylation	↓ birth weight, higher 1-year-weight and 3-year higher BMI in mothers with vitamin D deficiency	Clinical outcomes without clear methylation mediator [77].
25	Population-based	Mother/child pairs from SWS cohort (*n*= 230)	Maternal 25(OH)D serum level	↓ *RXRA* methylation in one CpG site	↑ Bone mineral content in 4-year-olds correlated with higher *RXRA* methylation	Suggested involvement of *RXRA* methylation changes in maternal 25(OH)D status effect on offspring bone mass [81].

↑ increase/upregulation ↓ decrease/downregulation.

**Table 6:** Summary of key findings on epigenomic effects of cholecalciferol supplementation from interventional studies.

No.	Study Type	Study Subject	Intervention/Exposure	Epigenetic Effect	Phenotypic Effect	Overall Conclusions
1	Prospective single-arm study	Adults (*n* = 50); whole blood DNA	1-year oral supplementation of 1200 IU cholecalciferol, B vitamins, calcium	No change in LINE-1 methylation	None	No global methylation effect in blood DNA [60].
2	Prospective single-arm study	Patients with IBD + COVID-19 infection (*n* = 32); extracellular vesicles (EVs)	Cholecalciferol 25–500 IU/month depending on 25(OH)D serum level	↑ miR30d-5p, miR150-5p, Let-7f-5p, and Let-7a-5p in the anti-SARS-CoV-2-positive + low 25(OH)D + cholecalciferol supplemented groups	Postulated regulation of oxidative stress and inflammation	Possible biomarkers for vitamin D response [134]
3	Prospective single-arm study	Healthy adults (65–79 years; Italians and Poles, *n* = 120)	Mediterranean diet + 400 IU cholecalciferol supplementation (1 year)	Trend toward ↓ DNAm age; significant in Polish female subgroup and epigenetically older participants at baseline	Not assessed	Modest subgroup-specific effect on epigenetic aging markers [67]
4	Non-randomized interventional study	Patients with mild cognitive impairment (*n* = 25) and control (*n* = 20)	Cholecalciferol and/or marine omega-3 fatty acid supplements (2 years)	↓ PhenoAge and GrimAge in mild cognitive impairment group	Not assessed	Cholecalciferol may be associated with a slower rate of epigenetic aging in patients with mild cognitive impairment [68]
5	RCT	Vitamin D-deficient young African Americans, overweight/obese (*n*= 51); leukocytes	Cholecalciferol monthly oral dose 18,000 IU/60,000 IU/120,000 IU/placebo for 16 weeks	↓ Horvath clock, non-conclusive for Hannum clock	Not assessed	Dose-dependent methylation response, including epigenetic aging markers [66]
6	RCT	Ultra marathon runners 36–40 years old (*n* = 20)	Cholecalciferol supplementation (10,000 IU/day) vs. control	↑ hsa-miR-155	↓ IL-1β ↑ TNF-α	High-dose cholecalciferol alters the inflammatory profile in high-performance athletes [132].
7	RCT	Men from couples about to undergo in vitro fertilization (*n* = 17); sperm	Dietary intervention: olive oil, cholecalciferol, and omega-3 fatty oils for 6 weeks (*n* = 8) vs. control (*n* = 9)	Altered expression of 3 tRFs, 15 miRNAs and 112 piRNAs	Targeted genes involved in fatty acid metabolism and transposable elements in the sperm genome.	Short-term combined supplementation induces broad non-coding RNA changes [140].
8	RCT	MASLD patients (*n* = 46)	Cholecalciferol supplementation 4000 IU/day (12 weeks) vs. placebo	↓ miR-21, miR-122	Improved liver, lipid, glucose metabolism markers and serum fibrogenic factors	Cholecalciferol supplementation improved metabolic dysfunction connected to MASLD [112].
9	RCT	Patients with prediabetes (*n* = 42)	Cholecalciferol supplementation 2000 IU/day (4 months) vs. placebo	↓ miR-7, miR-192 ↑ miR-152	MiR-152 negatively correlated with HbA1c, miR-192 positively correlated with fasting glucose	Cholecalciferol supplementation was associated with improvement in glucose metabolism [113].
10	RCT	Multiple sclerosis (MS) patients (*n* = 65); PBMCs	Cholecalciferol supplementation 1000 IU/day vs. 4000 IU/day for 4 months	↓ miR-155-5p expression	Correlation with *SARAF* gene (regulation of calcium release-activated channels)	Cholecalciferol supplementation reduces pro-inflammatory miRNA expression in MS patients independently of dose [133].
11	RCT	Male individuals with obesity (*n* = 77)	Cholecalciferol supplementation (20,000 IU or 40,000 IU/week, *n* = 40) vs. placebo (*n* = 77)	No effect on miRNA plasma profile after 12 months	Not assessed.	Study limited to 12 miRNAs; effects may be subtle, tissue-specific or context-dependent [103].
12	RCT	Mother/child pairs from MAVIDOS (*n*= 737)	Gestational cholecalciferol supplementation 1400 IU vs. 400 IU	No consistent *RXRA* methylation effect	No neonatal bone index differences; however, interaction between treatment effect and season of birth was observed	Inconsistent evidence [82]
13	RCT	Mother/infant pairs (*n* = 16); leukocytes from mothers at birth, 4–6 weeks of lactation and from infants at 4–6 weeks of life	Maternal cholecalciferol supplementation (3800 IU vs. 400 IU) during the study, including postpartum	Loci-specific methylation loss/gain in mothers and infants	Genes involved in metabolic, immune, and neurologic function, cadherin signaling, vascular/endothelial development	Methylation patterns might be a potential mechanism of adverse events during pregnancy and infant development [78]
14	RCT post hoc analysis	Mother/newborn pairs (*n* = 92); cord blood	Gestational cholecalciferol supplementation 4400 IU vs. 400 IU	↓ DNAmGAA (Knight’s and Bohlin clocks) in African American participants	GAA was associated with higher birth weight	Effect might reflect lower baseline 25(OH)D status among African American group [83]
15	RCT post hoc analysis	Women ≥ 55 years old from intervention group from CaMEWS trial (*n* = 446) with subgroup analysis of 36 extreme responders	Cholecalciferol 1100 IU/day supplementation + Calcium over 5-year period	↓ *CYP2R1* methylation; CpG-specific *CYP24A1* changes in the validation study; ↓ *CYP24A1* methylation in extreme responder group	Postulated influence on 25(OH)D serum level	DNA methylation levels of *CYP2R1* and *CYP24A1* might be involved in differences in response to cholecalciferol supplementation [102]

↑ increase/upregulation ↓ decrease/downregulation.

## Data Availability

No new data were created or analyzed in this study.

## Abbreviations

1,25(OH)_2_D—1,25-dihydroxycholecalciferol (calcitriol); 25(OH)D—25-hydroxycholecalciferol (calcifediol); APC—adenomatous polyposis coli; ART—aerobic-resistance training; AT—aerobic training; BAK1—BCL2 antagonist/killer 1; BMI—body mass index; BMP2K—BMP2 inducible kinase; BMC—bone mineral content; CD274 (PD-L1)—programmed death-ligand 1; CDKN1B (p27)—cyclin-dependent kinase inhibitor 1B; CDKI—cyclin-dependent kinase inhibitor; ceRNAs—competing endogenous RNAs; circRNAs—circular RNAs; CTCF—CCCTC-binding factor; COL4A1—collagen type IV alpha 1 chain; COX-2—cyclooxygenase-2; CpG—cytosine-phosphate-guanine; CRH—corticotropin-releasing hormone; CRP—C-reactive protein; CRC—colorectal cancer; CYP24A1—cytochrome P450 family 24 subfamily A member 1; DBP—vitamin D-binding protein; DHCR7/NADSYN1—7-dehydrocholesterol reductase/NAD synthetase 1; DENV-2—dengue virus type 2; DKK1—dickkopf WNT signaling pathway inhibitor 1; DNAm age—DNA methylation age; DNAmAA—DNA methylation age acceleration; DNAmGA—gestational DNA methylation age; DNMTs—DNA methyltransferases; DR3—direct repeat with a three-nucleotide spacer; EAE—experimental autoimmune encephalomyelitis; eNOS—endothelial nitric oxide synthase; EPCs—endothelial progenitor cells; EPHX1—epoxide hydrolase 1; EVs—extracellular vesicles; FOXN2-as—FOXN2 antisense RNA; GC—group-specific component (vitamin D binding protein); H19—H19 imprinted maternally expressed transcript; H19-as—H19 antisense RNA; HATs—histone acetyltransferases; HDACs—histone deacetylases; HFD—high-fat diet; HNC/HNSCC—head and neck (squamous) cancer; HNRNPH1—heterogeneous nuclear ribonucleoprotein H1; IGF1—insulin-like growth factor 1; IGFBP3—insulin-like growth factor binding protein 3; IFN-I—type I interferon; IL—interleukins; JARID2—jumonji and AT-rich interaction domain containing 2; JMJD5/JMJD6—jumonji domain-containing protein 5/6; KDM2B/KDM4A/KDM4C/KDM4D/KDM5A/KDM5B—lysine demethylases; Kcnq1ot1—KCNQ1 opposite strand/antisense transcript 1; LFD—low-fat diet; LINE-1—long interspersed nuclear element-1; lincRNA-p21—long intergenic non-protein coding RNA p21; lncRNAs—long non-coding RNAs; LPS—lipopolysaccharide; LSD1/LSD2—lysine-specific demethylase 1/2; MAPK—mitogen-activated protein kinase; MASLD—metabolic dysfunction-associated steatotic liver disease; miRNAs/miR—microRNAs; MI—myocardial infarction; MSCs—mesenchymal stem cells; MS—multiple sclerosis; mHOTAIR—HOTAIR (mouse); NADSYN1/DHCR7—NAD synthetase 1/7-dehydrocholesterol reductase; NELL2—neural EGFL like 2; NF-κB—nuclear factor kappa-light-chain-enhancer of activated B cells; NFAT5—nuclear factor of activated T cells 5; OGN—osteoglycin; Panx1—pannexin-1; PBMCs—peripheral blood mononuclear cells; PCNA—proliferating cell nuclear antigen; PDCD1LG2—programmed cell death 1 ligand 2; PI3K—phosphoinositide 3-kinase; piRNAs—PIWI-interacting RNAs; RCT—randomized controlled trial; RERE—arginine-glutamic acid dipeptide repeats; RXR/RXRA—retinoid X receptor/retinoid X receptor alpha; SARAF—store-operated calcium entry-associated regulatory factor; SFRP2—secreted frizzled-related protein 2; SLC25A24—solute carrier family 25 member 24; SMAD—SMAD family proteins; SMARCD2—SWI/SNF related, matrix associated, actin dependent regulator of chromatin subfamily D member 2; SNRPN—small nuclear ribonucleoprotein polypeptide N; SPN—sialophorin (CD43); TADs—topologically associating domains; TET2—ten-eleven translocation protein 2; TGF-β—transforming growth factor beta; TLR9—toll-like receptor 9; TNF-α—tumor necrosis factor alpha; tRFs—tRNA-derived fragments; TSS—transcription start site; VDR—vitamin D receptor; VDREs—vitamin D response elements; VEGF—vascular endothelial growth factor.
